# Discrete microfluidics for the isolation of circulating tumor cell subpopulations targeting fibroblast activation protein alpha and epithelial cell adhesion molecule

**DOI:** 10.1038/s41698-017-0028-8

**Published:** 2017-07-25

**Authors:** Małgorzata A. Witek, Rachel D. Aufforth, Hong Wang, Joyce W. Kamande, Joshua M. Jackson, Swathi R. Pullagurla, Mateusz L. Hupert, Jerry Usary, Weiya Z. Wysham, Dawud Hilliard, Stephanie Montgomery, Victoria Bae-Jump, Lisa A. Carey, Paola A. Gehrig, Matthew I. Milowsky, Charles M. Perou, John T. Soper, Young E. Whang, Jen Jen Yeh, George Martin, Steven A. Soper

**Affiliations:** 1 Department of Chemistry, The University of Kansas, Lawrence, KS 66047 USA; 2 Center of Biomodular Multiscale Systems for Precision Medicine, The University of Kansas, Lawrence, KS 66047 USA; 3 Department of Biomedical Engineering, The University of North Carolina, Chapel Hill, NC 27599 USA; 4 Department of Surgery, The University of North Carolina, Chapel Hill, NC 27599 USA; 5 BioFluidica, Inc., c/o Carolina Kick-Start, 321 Bondurant Hall, Chapel Hill, NC27599 USA; 6 Department of Genetics, The University of North Carolina, Chapel Hill, NC 27599 USA; 7 Lineberger Comprehensive Cancer Center, The University of North Carolina, Chapel Hill, NC 27599 USA; 8 Division of Gynecologic Oncology, Department of Obstetrics and Gynecology, UNC-Chapel Hill, NC 27599 USA; 9 Animal Histopathology Core, The University of North Carolina, Chapel Hill, NC 27599 USA; 10 Department of Pathology and Laboratory Medicine, The University of North Carolina, Chapel Hill, NC 27599 USA; 11 Department of Medicine, Division of Hematology and Oncology, The University of North Carolina, Chapel Hill, NC 27599 USA; 12 Department of Pharmacology, The University of North Carolina, Chapel Hill, NC 27599 USA; 13 Roche, Pleasanton, CA 94588 USA; 14 BioEngineering Program, The University of Kansas, Lawrence, KS 66047 USA; 15 Department of Mechanical Engineering, The University of Kansas, Lawrence, KS 66047 USA; 16 Ulsan National Institute of Science and Technology, Ulsan, Republic of Korea

## Abstract

Circulating tumor cells consist of phenotypically distinct subpopulations that originate from the tumor microenvironment. We report a circulating tumor cell dual selection assay that uses discrete microfluidics to select circulating tumor cell subpopulations from a single blood sample; circulating tumor cells expressing the established marker epithelial cell adhesion molecule and a new marker, fibroblast activation protein alpha, were evaluated. Both circulating tumor cell subpopulations were detected in metastatic ovarian, colorectal, prostate, breast, and pancreatic cancer patients and 90% of the isolated circulating tumor cells did not co-express both antigens. Clinical sensitivities of 100% showed substantial improvement compared to epithelial cell adhesion molecule selection alone. Owing to high purity (>80%) of the selected circulating tumor cells, molecular analysis of both circulating tumor cell subpopulations was carried out in bulk, including next generation sequencing, mutation analysis, and gene expression. Results suggested fibroblast activation protein alpha and epithelial cell adhesion molecule circulating tumor cells are distinct subpopulations and the use of these in concert can provide information needed to navigate through cancer disease management challenges.

## Diagnostics: Dual selection improves circulating tumor cell assay

Using two selection markers, instead of one, can improve the sensitivity of a blood test for circulating tumor cells (CTCs). Steven A. Soper from the University of Kansas in Lawrence, USA, and colleagues analyzed blood samples from patients with cancers of the pancreas, colon, breast, ovaries and prostate, as well blood from healthy donors and those with benign disease. They ran each patient’s blood through two microfluidic devices, one chip targeting the established marker epithelial cell adhesion molecule (EpCAM) and another targeting a new marker, fibroblast activation protein alpha (FAPα). Doing so detected CTCs in nearly all the cancer patients—a substantial improvement over testing for EpCAM CTCs alone. Molecular and genetic analyses of the CTCs showed that those expressing EpCAM and FAPα are distinct subpopulations, and testing for both could prove valuable for clinical management.

## Introduction

Methods relying on anti-epithelial cell adhesion molecule (EpCAM) for positive affinity-selection of circulating tumor cells (CTCs) has been cleared by the Food and Drug Administration (FDA) for metastatic breast, prostate, and colorectal cancers; however, enumeration of EpCAM(+) CTCs alone has demonstrated modest clinical sensitivity.^1^ EpCAM-bearing CTCs may not be the only “players” in cancer progression. For example, CTCs undergoing epithelial-to-mesenchymal transitions^2^ lose epithelial antigens due to phenotypic plasticity. Additionally, the tumor microenvironment is composed of phenotypically distinct cells that may be involved in disease progression.^3^ Therefore, for CTC selection it becomes necessary to consider orthogonal markers in combination with the epithelial ones to improve clinical sensitivity, patient stratification, disease recurrence monitoring, and/or therapeutic guidance.

The use of multiple affinity-selection markers has been attempted with a combination of monoclonal antibodies, mAbs (i.e., EpCAM plus TROP-2, HER-2, and CD44).^4^ In metastatic cancer patients, this strategy recovered EpCAM-negative cells that were cytokeratin (CK)-positive, contrasting with the classical CTC definition of EpCAM+/CK+/CD45−^4^. While recovering CTCs on mixed monolayers of mAbs has been reported, subpopulations cannot be independently interrogated unless elaborate single-cell analysis is employed. Additionally, because CTC affinity-selection depends upon the mAb surface concentration, mixed monolayers can reduce recovery, especially when CTCs express low antigen levels. Positive CTC selection markers have included prostate-specific membrane antigen (PSMA), chemokine receptors, CD133, VCAM-1, MCAM (CD146), ICAM-1, CEA, HER-2, N-cadherin (CDH2)/O-cadherin (CDH11), and MUC1.^5–7^ Some of these antigens target only a particular cancer (i.e., PSMA) or lack cancer-specificity (CD133, VCAM-1, ICAM-1) as hematopoietic/endothelial/benign cells also expressed these antigens,^8–11^ producing low CTC purity and confounding clinical interpretations of the data. Other markers (MUC1) are co-expressed with EpCAM and thus provide modest improvement in clinical sensitivity.^6^

We report a CTC selection strategy that uses serially connected microfluidic chips (i.e., discrete microfluidics) to affinity-select two CTC subpopulations expressing EpCAM and fibroblast activation protein alpha (FAPα).^12^ FAPα expression has been observed in >90% of human epithelial cancers and has been associated with mesenchymal characteristics and cell invasion of the extracellular matrix.^13^ Our choice for investigating FAPα CTCs was further guided by data from the Human Protein Atlas, which indicated mutually independent, orthogonal expression of FAPα and EpCAM across many cancer cell lines (Supplementary Fig. S1). FAPα has been identified via staining in CTCs that invade a cell adhesion matrix (CAM),^14^ but to date, affinity-selection of FAPα and EpCAM-bearing CTC subpopulations for enumeration from clinical samples, molecular profiling, and longitudinal surveillance has not been undertaken.

We hypothesized that FAPα can be used as an additional marker for selecting a phenotypically distinct CTC subpopulation with respect to a CTC subpopulation that expresses EpCAM. In addition, parsing these subpopulations into different fractions could provide molecular characteristics of distinct cancer cell phenotypes that could be useful in better predicting clinical outcomes.

## Results

### Microfluidic CTC selection strategy

**Fig. 1:**
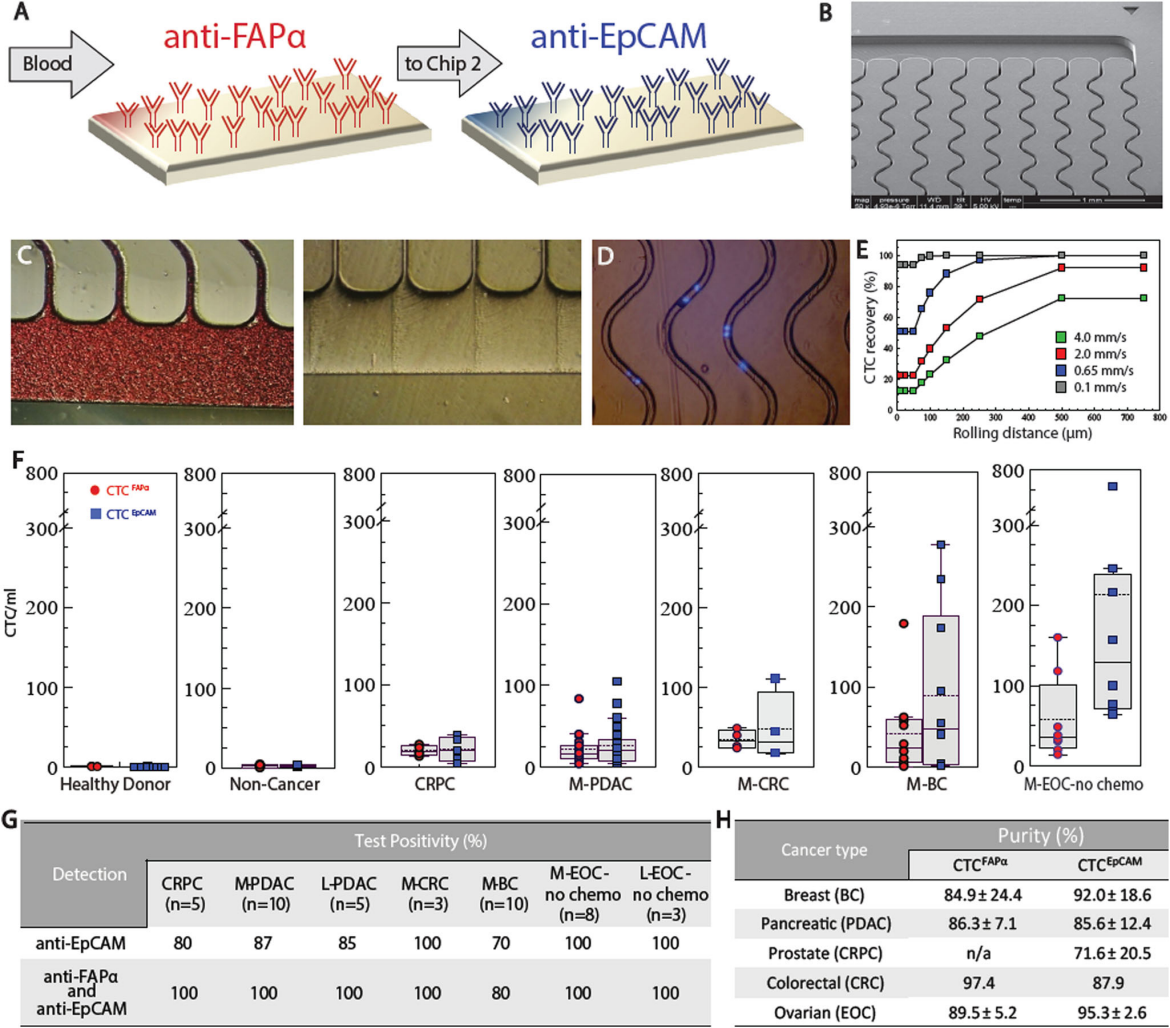
Sinusoidal microfluidic device used in the study and summary of clinical results. **a** Schematic of the dual selection strategy using mAbs directed against FAPα and EpCAM cell-surface antigens. **b** SEM of the CTC selection microfluidic device. **c** Optical micrographs of the CTC selection microchip filled with whole blood, and the chip after rinsing with buffer. **d** An image (5×) of DAPI-stained Hs578T cells isolated within the channels of the microfluidic device. **e** Simulation of CTC recovery from blood at different translational velocities as a function of cell rolling distance along the mAb decorated surface. **f** Box plots for CTCs isolated from the blood of healthy donors, patients with non-cancerous disease, CRPC, M- PDAC, M-CRC, M-BC, and M-EOC. CTC counts were normalized to 1 ml of blood. **g** Test positivity in cancer patients’ blood using the single EpCAM approach and the dual selection strategy (test positivity based on the CTC^FAPα^ and/or CTC^EpCAM^ counts exceeding a level that was 3× SD for counts from non-cancer patients)

To demonstrate the utility of the dual selection assay for CTCs in this study, we employed sinusoidal microfluidics for CTC affinity isolation (Fig. 1a–c). The microfluidic chips process whole, unfractionated, and unfixed blood and use sinusoidal microchannels (Fig. 1b) to encourage interactions between flowing CTCs and mAbs decorated on the device’s surfaces (Fig. 1c, d) for affinity isolation. In previous reports, we have characterized the sinusoidal technology for its operating principles and performance,^15–20^ isolated EpCAM+ CTCs in patients with localized and metastatic pancreatic ductal adenocarcinoma (L-PDAC and M-PDAC, respectively),^17^ metastatic epithelial ovarian cancer (M-EOC),^18^ and patient-derived xenograft pancreatic ductal adenocarcinoma (PDAC) mouse models.^20, 21^ We also applied this technology for residual disease evaluation in multiple myeloma^18^ and acute myeloid leukemia^19^ patients, as well as T-cells and neutrophil isolation for stroke diagnostics.^22^ The sinusoidal technology offers high CTC recovery from clinical samples while achieving exquisite purity that enabled much of the molecular profiling reported herein (Fig. 1g).

In this study, we selected both FAPα+ and EpCAM+ cells from a single blood sample by arranging two microfluidic devices in series, one chip targeting FAPα+ and the other EpCAM+ cells (Fig. 1a, b). The CTC selection devices were made from cyclic olefin copolymer (COC) via hot embossing, and each device’s surfaces were covalently decorated with a single mAb type (see Methods).^17, 20^ Blood entered the first CTC selection device through a single inlet channel, passed through a parallel array of 50 sinusoidal mAb-laden selection channels at 2 mm/s (1.5 ml/h) (Fig. 1c),^15, 16^ and exited through a single outlet channel, which then fed the second device that was identical except for the identity of the selection mAb. After blood processing and washing, the chips could be disconnected so each CTC subpopulation could be interrogated independently, data which would have been obscured by immobilizing both mAbs in one device.

Several aspects of the sinusoidal architecture were optimized (125 µm radius of curvature, 25 µm width, and 150 µm depth) to maximize recovery, throughput, and purity (Fig. 1b, d).^15, 16, 23^ The CTC affinity-selection process can be separated into two parts, initiation of contact between a CTC and the mAb-coated surface and successful binding of the rolling CTC with surface-confined mAbs. For the first process, the sinusoidal architecture generated centrifugal forces (*F*_c_) to propel CTCs towards the mAb-coated channel walls with a magnitude that varies with cell diameter, density, and forward velocity (*V*). For a 16 µm CTC traveling at 2 mm/s, the resultant centrifugal velocity (*V*_c_) is 1.9 µm/s, four times greater than an 8 µm leukocyte. Channel width is critical to *F*_c_’s effectiveness. In 25-µm wide channels, only a 4.5 µm shift in position is needed for a 16 µm CTC to interact with the wall, and a 15 s residence time is provided to produce a *V*_c_ of 1.9 µm/s that helps facilitate CTC-mAb interactions.^16^

While *V*_c_ can be enhanced by increasing the cell’s forward velocity *V*, the trade-off is lower probability of successful binding of rolling CTCs and surface-bound mAbs. The binding dynamics of CTC microfluidic affinity-selection can be described by the Chang–Hammer model,^24^ (see Eq. 1) which balances mAb-antigen binding kinetics, the residence time of the traveling CTC near a mAb, and the number of antigens on a CTC, with recovery becoming less probable at very high linear velocities and low antigen expression.

1 $${P_R} = 1\, - \,1/{e^{\frac{{{N_R}L{k_f}}}{V}}}.$$

In Eq. 1, the probability (*P*_R_) of CTC recovery and the forward binding constant (*k*_f_) are a function of how often Ab-antigen interactions occur and how probable a given binding event is considering the balance of the Ab-antigen binding kinetics with the reaction time. Recovery should: (i) decrease as the cell’s velocity (*V*) is increased due to shorter reaction time, and (ii) increase with the surface density of antigens expressed on the CTC (*N*_R_). As the CTC rolls over the surface with increasing length (*L*), *P*_R_ increases and leads to higher recovery.

An aspect of the sinusoidal CTC chip that evolved from the Chang–Hammer model is related to long rolling distances of CTCs over the continuous microfluidic surface, which improves recovery by accumulating more potential binding events (Fig. 1e).^16^ This accumulative effect of long rolling distances in the sinusoidal architecture (>250 µm) is especially important to provide high recovery of CTCs with low antigen expression (limit of 700 molecules per 16 µm CTC under shear force)^19^ and enables operation at relatively high *V* (2 mm/s),^15, 16^ which maintains high throughput (1.5 ml/h) and generates high fluidic shear stress (13.3 dynes/cm^2^) that disrupts non-specific adsorption of leukocytes to the mAb-coated COC polymer surface and yields the sinusoidal technology’s uniquely high purity.^25^

### CTC recovery and orthogonality of the dual selection strategy

Two breast cancer cell lines, Hs578T and SKBR3, representing FAPα + CTCs (CTC^FAPα^) and EpCAM + CTCs (CTC^EpCAM^), respectively, were chosen to evaluate cell recovery and cross-reactivity using the dual selection strategy. These cell lines were characterized by multi-parameter flow cytometry, immunophenotyping, and mRNA gene expression (Supplementary Fig. S2A–C).

For Hs578T and SKBR3 cells spiked into healthy donors’ blood, the average recovery (±SD) were 75 ± 8% and 77 ± 2%, respectively. For comparison, the recovery of MCF-7 cells (higher expression of EpCAM than SKBR3) using the same architecture device and modification chemistry was 83 ± 5%.^17^ The purity of the selected CTC fractions seeded at ~100 cells/ml into healthy donors’ blood was 93 ± 3% (Hs578T) and 91 ± 4% (SKBR3). Additionally, the cross-reactivity of Hs578T cells on the anti-EpCAM selection chip was 4 ± 2% (*n* = 3), and SKBR3 cells on the anti-FAPα selection device was 8 ± 3% (*n* = 3).

We selected both FAPα+ and EpCAM+ cells from a single blood sample by arranging two microfluidic devices in series. The effect of the order in which the devices were positioned on CTC recovery was investigated; no preferential CTC isolation on the first chip was observed (Supplementary Table S1 **)**. Also, there was no statistical difference between the order of the chips. The dual selection strategy reproducibility for each chip produced an RSD of 25% (*n* = 33). For these studies, the FAPα selection chip was positioned first in the series.

### CTC dual selection from clinical samples

In a pilot clinical study, we analyzed blood from 11 healthy donors and 6 patients with benign disease (Supplementary Tables S2, S3), 5 L-PDAC, 10 M-PDAC, 3 localized colorectal cancer (L-CRC), 3 metastatic CRC (M-CRC), 10 metastatic breast ductal carcinoma (M-BC), 8 metastatic chemotherapy naïve EOC (M-EOC-no-chemo), 5 metastatic EOC that received neo-adjuvant chemotherapy (M-EOC-chemo), 3 localized chemotherapy-naïve EOC (L-EOC-no-chemo), and 5 castration resistant prostate cancer (CRPC) patients (Supplementary Tables S4–S8). Each CTC subpopulation was enumerated independently. CTCs were stained for CKs, CD45, and 4′,6-diamidino-2-phenylindole (DAPI) or counted using an impedance sensor following enzymatic release from the capture surface.^17^ Impedance sensing is a detection strategy of single cells that obviates the need for staining, which may interfere with the molecular analyses.^15, 17^ For the present study, following CTC isolation, cells were released from the device using trypsin and infused between electrodes operated at 40 kHz; each cell generates a detectable voltage pulse that correlates with cell size. An example of an impedance trace for CTCs is presented in Supplementary Fig. S3. We compared both CTC^FAPα^ and CTC^EpCAM^ counts obtained via staining and impedance sensing by performing duplicate analyses for randomly selected samples (Supplementary Table S9). The CTC counts obtained by both methods were similar, and any differences most likely reflect Poisson statistics.

In blood from healthy donors, no CTC^FAPα^ or CTC^EpCAM^ were detected. The mean for CTC^FAPα^ and CTC^EpCAM^ in patients with non-cancer disease was 1.8/ml and 2.6/ml, respectively (Supplementary Table S10). CTC test positivity and test specificities were determined by establishing a threshold value based on 3 × SD for cells detected in healthy and non-cancer disease patients. The test specificity at this threshold was 100% (*n* = 17). Dual CTC selection provided 100% test positivity for patients with all malignancies but M-BC (Fig. 1h), which yielded 80%.

For cancer patient samples, the number of CTC^FAPα^ and CTC^EpCAM^ varied with the disease type (Fig. 1f). Pairwise statistical analysis showed a significant difference between CTCs detected in cancer patients and healthy donors or patients with non-cancer disease (Supplementary Table S11).

CTC^FAPα^ were most prevalent in M-CRC (26–49/ml), while CTC^EpCAM^ were most abundant in chemotherapy-naïve M-EOC (65–680/ml; Supplementary Table S10). In M-EOC, the median CTC^EpCAM^ was higher for chemotherapy-naïve patients compared to patients undergoing chemotherapy (129/ml vs. 42/ml, *p* = 0.007). Conversely, there was no change observed in CTC^FAPα^ counts between these two groups (36 vs. 32/ml). CTC^FAPα^ numbers were 2 × lower in L-EOC-no-chemo patients (18/ml; Supplementary Fig. S4A, B).

The recoveries of CTCs evaluated from clinical samples, determined using the “self-referencing” method (see SI),^18^ for randomly selected samples were found to be 79 ± 7% (*n* = 3) and 87 ± 2% (*n* = 3) for CTC^FAPα^ and CTC^EpCAM^, respectively. The difference in the clinical recovery arises from dissimilarities in the level of antigen expression and cell size within a CTC subpopulation. The purity determined for each individual selection bed, defined as [CTCs/(CTCs + leukocytes)], are reported in Fig. 1h with WBCs counts reported in Supplementary Tables S14–S19.

**Fig. 2:**
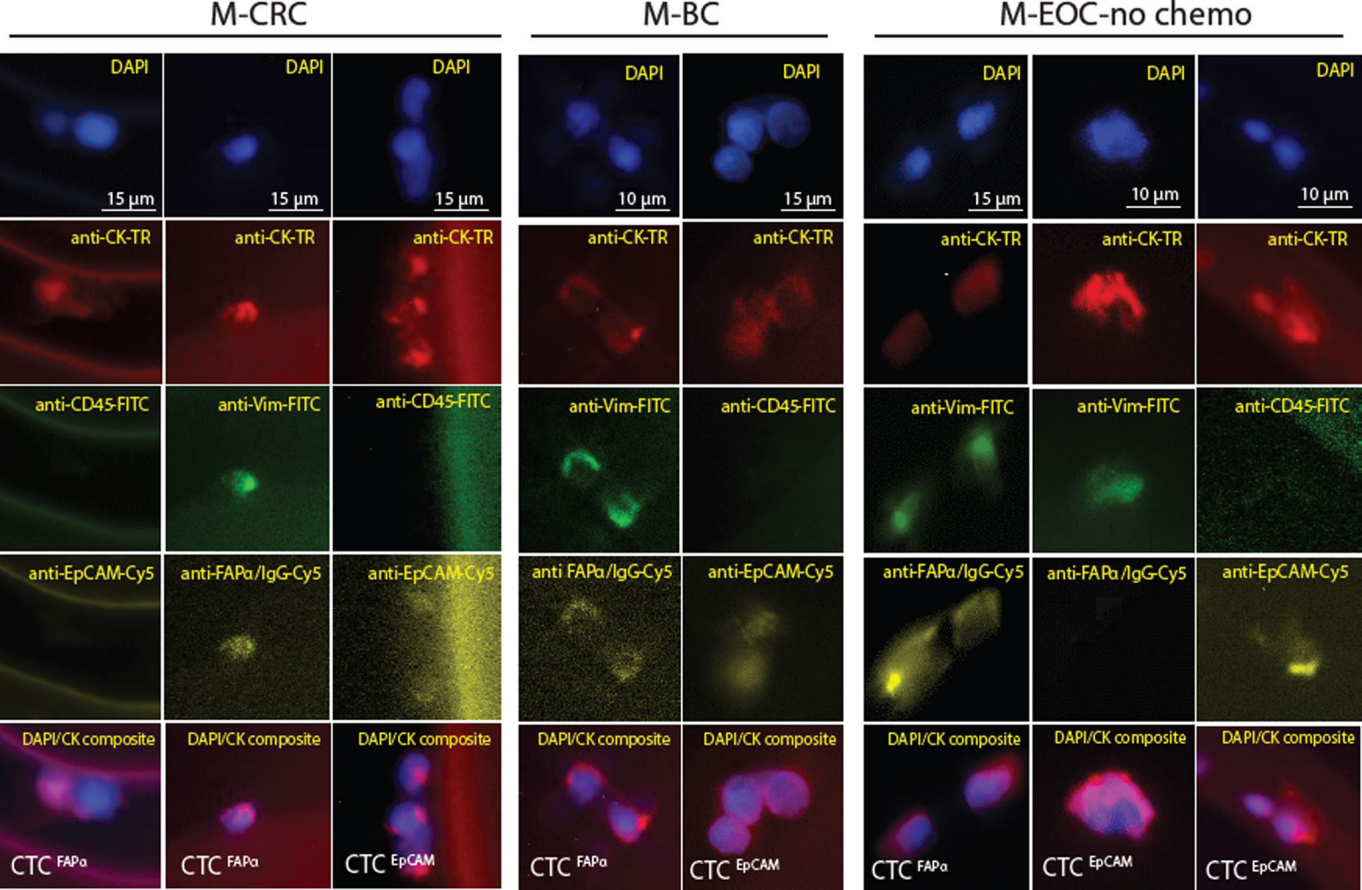
Phenotyping analysis in fluorescence microscopy. Images (40×) of CTC^FAPα^ and CTC^EpCAM^ isolated using the sinusoidal microfluidic chips and stained with a panel of markers: DAPI, anti-pan-CK-TR, anti-CD45-FITC, anti-VIM-FITC, anti-EpCAM-Cy5, and anti-FAPα-Cy5

Fluorescence images of CTC^FAPα^ and CTC^EpCAM^ isolated from M-CRC, M-BC, and M-EOC-no-chemo (Fig. 2) showed that both subpopulations displayed characteristics attributed to a CTC (i.e., large nuclear/cytoplasm ratio). However, differences in morphological features between CTC^FAPα^ and CTC^EpCAM^ were not conclusive mainly due to the nature of the affinity-based selection process, which can change the appearance of cells upon antigen–mAb binding to solid surfaces in the presence of shear forces. No CTCs were identified that were triple stained (DAPI+, CK+, CD45+) or showed only nuclear staining.

### Immunophenotyping of CTC subpopulations in clinical samples

**Fig. 3:**
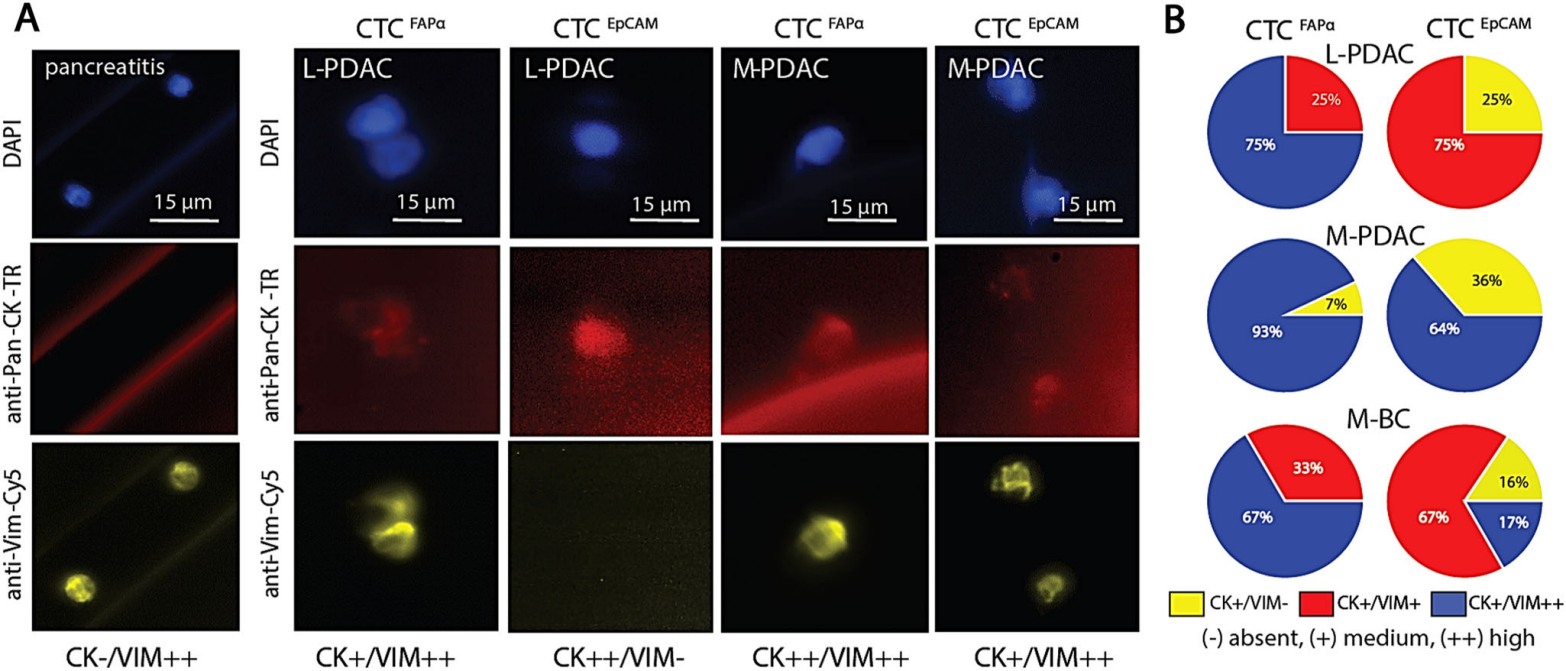
CTC phenotyping. **a** Fluorescence micrographs of cells isolated from a patient diagnosed with pancreatitis, and CTC^FAPα^ and CTC^EpCAM^ isolated from L/M-PDAC patients. All cells stained negative for CD45. **b** Immunophenotyping results of CTC^FAPα^ and CTC^EpCAM^. The pie charts show the percent of CTCs with pan-CK and/or VIM expression for L-PDAC patient #66, M-PDAC patient #25, and M-BC patient #5

Selected CTCs were immunophenotyped for expression of CD45, pan-CK (epithelial marker), and VIM (mesenchymal marker). The fluorescence intensity was normalized (see Methods) and CTCs were classified as showing no (−), medium (+) or high (++) expression of the appropriate marker. Examples of different phenotypes are shown in Fig. 3. Figure 3a shows two FAPα+ cells isolated from a pancreatitis patient. These rare cells were CK−/VIM++/CD45− with a low nuclear-to-cytoplasmic ratio and were classified as circulating fibroblasts and not CTC^FAPα^.

In L/M-PDAC patients, most CTC^FAPα^ were VIM++ and CK+ (Fig. 3b). CTC^EpCAM^ with this phenotype were found in M-PDAC, but not in L-PDAC patients. In L-PDAC, the CTC^EpCAM^ dominating fraction equally expressed VIM and CK with some cells VIM- and CK++.

For a triple negative M-BC patient, the majority of CTC^FAPα^ showed VIM++ and CK+ with the remaining CTCs equally expressed CK and VIM (Fig. 3b). CTC^EpCAM^ showed all phenotype combinations.

These results indicated the presence of different phenotypes among CTC^FAPα^ and CTC^EpCAM^; VIM++ and CK+ implied a mesenchymal type, VIM− and CK++ an epithelial one, and a third phenotype showing co-expression of CK and VIM suggested a cell undergoing epithelial–mesenchymal transition (EMT).^2^

### Longitudinal tracking of PDAC patients

**Fig. 4:**
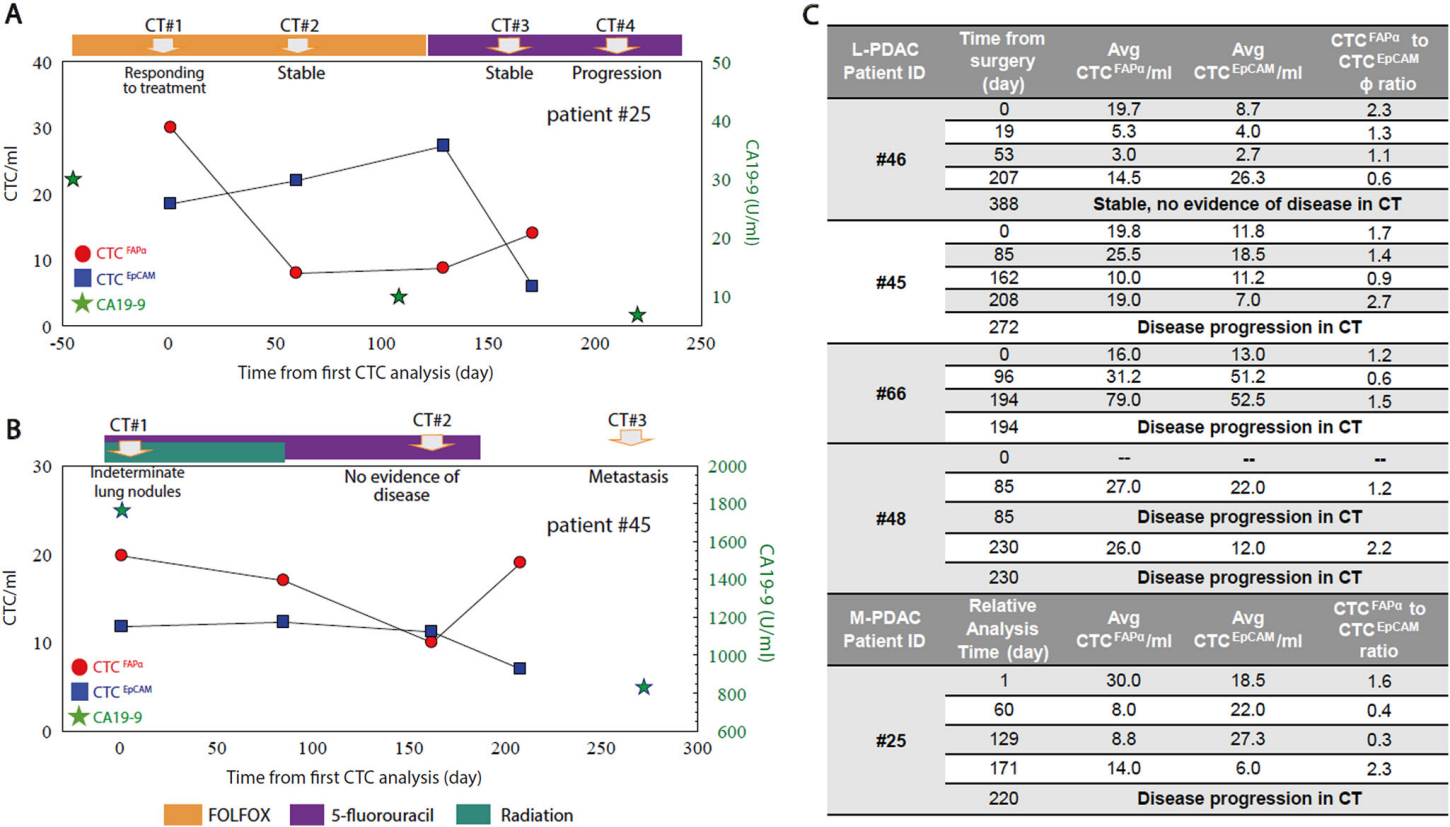
Longitudinal tracking of CTC^FAPα^ and CTC^EpCAM^ numbers in the blood of PDAC patients. **a** M-PDAC patient #25. The first CTC analysis was performed during second-line therapy (*t* = 0). **b** L-PDAC patient #45. The first CTC analysis in this case was performed pre-operatively on the day of surgery (*t* = 0). CA19-9 measurements (*green stars*) are shown when available. CTC^FAPα^ = *red dots*, and CTC^EpCAM^ = *blue squares*. Points are connected for ease of visualization, but do not represent any type of functional relationship between the individual data points. **c** A summary of all patients tested in this longitudinal study

Figure 4a–c shows longitudinal tracking results for five PDACpatients. The first CTC test for 3/5 of these patients was obtained preoperatively on the day of surgery. It appeared that CTC^FAPα^ were the dominating population at that time as indicated by CTC^FAPα^/CTC^EpCAM^ ratio (defined as *ϕ*) ranging between 1.2 and 2.3 (Fig. 4b, c).

Figure 4a shows longitudinal tracking of M-PDAC patient #25. Levels of CTC^FAPα^ decreased 60 days after the initial analysis with no significant change observed on day 129. CTC^EpCAM^ increased slightly; computed tomography (CT) imaging was consistent with stable disease over this time period and *ϕ* was 0.3. However, CTC analysis on day 171 showed a nearly 2-fold increase in CTC^FAPα^ and a significant drop in the CTC^EpCAM^ burden, with *ϕ* equal to 2.3. This patient’s disease later showed progression by CT imaging. CA19-9 levels were low and continually decreased over the entire testing period (normal < 35 U/ml).

Figure 4b shows results for L-PDAC patient #45. Pre-operative CTC^FAPα^ was 20/ml; CTC^EpCAM^ was 12/ml (*ϕ* = 1.7); and CA19-9 was 1764 U/ml. On day 162, the CTC burden was ~10/ml for both subpopulations (*ϕ* = 0.6), and CT imaging was not definitive for disease recurrence. On day 208, CTC^FAPα^ counts increased to 20/ml, while CTC^EpCAM^ were 7/ml (*ϕ* = 2.7). CT imaging for this patient thereafter showed metastatic disease. CA 19-9 levels decreased 2-fold from the pre-operative level but remained high at 831 U/ml. In PDAC pt#45 and #25 CA 19-9 levels did not correlate with disease progression as determined by CT.

In L-PDAC patient #48 on day 85, both CT scan and CTC analysis were performed. Both CTC subpopulations were enumerated (*ϕ* = 1.2), and the results of CT imaging indicated metastatic disease. A subsequent CTC test administered on day 230 showed similar CTC^FAPα^ burden (26/ml) and a decrease in CTC^EpCAM^ numbers (*ϕ* = 2.2, Fig. 4c), and CT imaging determined disease progression.

In L-PDAC patient #66, the *ϕ* was 1.2 on the day of surgery. It decreased to 0.6 on day 96 following surgery, but increased again to 1.5 on day 194. CTC testing on day 194 detected the same burden of CTC^EpCAM^ (~51/ml) as found on day 96, but a 2.5-fold increase in CTC^FAPα^ burden (31 v 79 CTC^FAPα^/ml) was observed (Fig. 4c). At that time (day 194), disease progression was determined via CT imaging.

In the fifth patient tested in the longitudinal study (patient #46), the *ϕ* was 2.3 on the day of surgery, indicating a dominant CTC^FAPα^ subpopulation. Nineteen and 53 days following surgery, post-operative chemotherapy and radiation, the CTC burden was low as only 3–5 CTC/ml were detected for both subpopulations. When the CTC test was performed at day 207, the counts for both CTC subpopulations increased (14/ml for CTC^FAPα^ and 26/ml CTC^EpCAM^), with CTC^EpCAM^ being the dominating population (*ϕ* = 0.6, Fig. 4c). About a year following surgery, this patient’s disease was classified as stable by CT.

For all aforementioned PDAC patients, we analyzed CTC results for which clinical notations were available: (i) samples acquired pre-operatively (localized disease), CT imaging indicating (ii) stable disease or (iii) metastasis (Supplementary Fig. S4). For this data set, the tandem analysis of both CTC^FAPα^ and CTC^EpCAM^ subpopulations appeared to be a better indicator of PDAC disease state than the analysis of either subpopulation alone (Fig. 4c and Supplementary Fig. S5).

### CTC next generation sequencing (NGS) and mutation detection using the polymerase chain reaction/ligase detection reaction (PCR/LDR)

When isolated CTC fractions are of low purity, single-cell picking must be performed to eliminate wild type background. Given the high purity afforded by the sinusoidal microfluidic, we sought to obviate single cell picking and release CTCs in bulk from the microfluidic chip, and performing whole genome amplification (WGA) and NGS on the bulk affinity selected CTC subpopulations. We surveyed both CTC subpopulations isolated from a chemotherapy-naïve L-EOC patient (CTC^FAPα^ = 105, CTC^EpCAM^ = 717). Deep read depths (9900–65,000) allowed for high fidelity mutation detection. The CTC^FAPα^ and CTC^EpCAM^ gDNA contained the same missense somatic mutations in *TP53* and *CDH1* genes and other SNPs, suggesting these CTCs had the same origin (Supplementary Table S12).

**Fig. 5:**
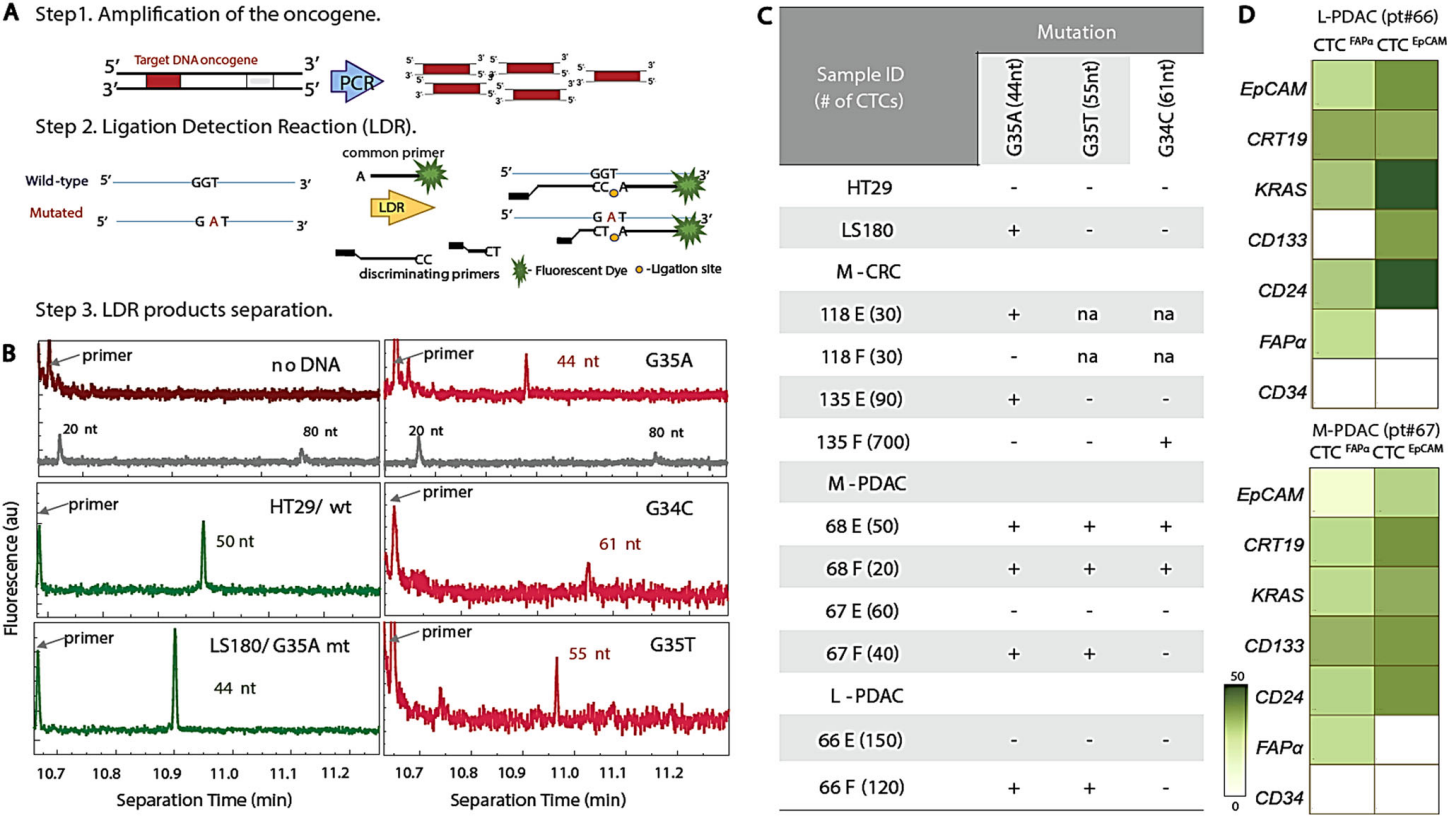
*KRAS* mutation detection. **a** Schematic of the polymerase chain reaction/ligase detection reaction (PCR/LDR) assay. **b** Electropherograms of LDR products for: No gDNA; HT29 wt35 (50 nt); LS180 G35A (44 nt); M-PDAC CTC^FAPα^ G35A (44 nt), CTC^EpCAM^ G34C (61 nt); and CTC^FAPα^ G35T (55 nt). The *gray trace* shows the DNA markers. The fluorescence intensity values are arbitrary. **c** Table summarizing PCR/LDR results for HT29 and LS180 cell lines, M-CRC, L-CRC, M-PDAC, and L-PDAC CTCs. **d** RT-qPCR gene expression profiles for L-PDAC patient #66 and M-PDAC patient #67

We also targeted *KRAS* mutations in CTCs using PCR/LDR (see Methods), a sensitive method due to dual amplification, to identify mutations in low copy numbers of DNA (Fig. 5a).^26^ By designing different length discriminating and common-fluorescently labeled primers, the LDR products differed in size depending on the specific *KRAS* mutation (Supplementary Table S13). LDR products were detected by capillary gel electrophoresis (Fig. 5b). gDNA from cell lines of known *KRAS* genotype (HT29—wild-type (wt) and LS180—mutated (mt) G35A) provided controls. HT29 gDNA showed peaks corresponding to 50 and 67 nt fragments indicating wt exon 1 codon 12 (wt35 and wt34, Supplementary Table S13), while LS180 gDNA showed an additional product of 44 nt (Fig. 5b) indicating mt G35A in this codon in agreement with the literature.^27^ LDR reactions without gDNA showed no products (Fig. 5b).

CTC subpopulations from one M-CRC, L-CRC, L-PDAC and two M-PDAC samples were independently genotyped (Fig. 5c). Tumor tissue was not available for testing.

The prevalence of *KRAS* mutations in PDAC is nearly ubiquitous and represents the earliest genetic alteration in this disease.^28^ In M-PDAC patient #68, both subpopulations showed three mutations in *KRAS* (Fig. 5b). However, in patients #66 and #67, CTC^EpCAM^ were not mutated, whereas CTC^FAPα^ showed G35A and G35T *KRAS* mutations. Multiple mutations are indicative of cancer cell aneuploidy, and this “polyclonality” of *KRAS* SNPs is a common feature in PDAC patients.^29^

For CRC patients, *KRAS* mutations are often found in codon 12 (80%), most frequently G35A and G35T.^30^ CTC^FAPα^ from M-CRC patient #118 was wt *KRAS*; however, CTC^EpCAM^ showed a G35A mutation. In L-CRC patient #135, we detected mt G34C in CTC^FAPα^ and mt G35A in CTC^EpCAM^ (Fig. 5c).

### Gene expression analysis of FAPα and EpCAM CTCs

While the molecular profiling of CTC was performed to obtain information on orthogonality or dissimilarity of evaluated CTC subpopulations, these data will demonstrate the translational capacity and clinical utility of molecular profiling CTCs isolated using the sinusoidal microfluidic device.

We evaluated possible mRNA expression changes due to microfluidic isolation using cell lines. Relative expression of mRNA for selected genes assessed for Hs578T and SKBR3 cells harvested from culture and affinity isolated on a microfluidic chip indicated no significant differences for the tested genes (Supplementary Fig. S6), indicating no obvious influence of the affinity selection process on mRNA expression.

CTC subpopulations were tested for their mRNA expression in five M-PDAC and two M-CRC patients (Fig. 5d, e and Supplementary Fig. S7). Gene expression patterns differed between CTC^FAPα^ and CTC^EpCAM^ subpopulations and were distinct from the patient’s T cells and buffy coat. *EpCAM* mRNA expression for the CTC^EpCAM^ subpopulation was 10-fold higher than CTC^FAPα^ for both cancer types, and *FAPα* mRNA was not found in the CTC^EpCAM^ subpopulation. Both results agreed with immunophenotyping; when CTCs were stained with fluorescently-labeled anti-EpCAM mAb, 89 ± 11% of CTC^EpCAM^ and 12 ± 6% CTC^FAPα^ had detectable EpCAM. *FAPα* mRNA expression was exclusively observed in CTC^FAPα^ but was rather low because the FAPα protein is a product of alternative splicing of ten different mRNAs. (http://www.ncbi.nlm.nih.gov/IEB/Research/Acembly/av.cgi?db=human&c=Gene&l=FAP) When two variants were tested in the Hs578T cell line, both *FAPα* mRNA were observed (Supplementary Fig. S2C). *VIM* mRNA was expressed higher in CTC^FAPα^ than CTC^EpCAM^ in M-PDAC; however, *VIM* expression was high in both subpopulations in M-CRC. mRNA expression profiling included stem cell markers (*CD133, CD24, and CD44)*. In M-PDAC, *CD133, CD24* mRNA was highly expressed in both subpopulations, with *CD44* showing expression only in CTC^EpCAM^. Both subpopulations of CTCs in M-CRC showed expression of *CD24* but lacked *CD44* and *CD133* (Supplementary Fig. S7A).

Both CTC subpopulations from M-PDAC and M-CRC patients lacked *CD34* mRNA, suggesting absence of endothelial cell character and no significant contamination from hematopoietic cells. *CD34* mRNA was expressed, as expected, in the M-PDAC buffy coat (Supplementary Fig. S7A).

*KRAS* mRNA in the CTC^EpCAM^ subpopulation was highly expressed when compared to CTC^FAPα^ for M-CRC (Supplementary Fig. S7A), which contrasted to M-PDAC. However, when we evaluated expression in individual patients (Fig. 5d), we observed that the wt *KRAS* gene in CTC^EpCAM^ was overexpressed, while for the mutated *KRAS* in CTC^FAPα^, expression was 10-fold lower. Similar observations were made for the L-PDAC patient. Overexpression of wt *KRAS* suggests activation of downstream signaling pathways.^31^

We tested *PSA* and *PSMA* mRNA gene expression in a CRPC patient (Supplementary Fig. S7B). *PSA* and *PSMA* mRNA were expressed in both CTC^FAPα^ and CTC^EpCAM^, suggesting these cells originated from the prostate tumor environment as *PSMA* and *PSA* mRNA expression is observed in normal prostate, hyperplastic, and invasive prostate carcinomas.^32^ CTC^FAPα^ were also stained with a fluorescently-labeled PSMA mAb, which confirmed the presence of this protein.

### CTC isolation from PDX: Do CTC^FAPα^ originate from human tumor or mouse-activated stroma?

FAPα is considered a marker of cancer-associated fibroblasts (CAFs), but it is also expressed by pericytes, fibrocytes, or fibroblasts during wound healing.^33^ CAFs or circulating fibroblasts are typically identified as expressing FAPα/SMAα/VIM but lacking CK and CD45,^34^ and are genetically stable.^33^ Fibrocytes detected in tumor stroma or bone marrow are FAPα+/CD34+/CD45+ while the FAPα+/ CD34+/CD45− phenotype suggests a mesenchymal stem cell.^35, 36^ Isolated CTC^FAPα^ expressed VIM, CK, but no CD45 (phenotyping and gene expression) and were CD34− as determined by mRNA expression (i.e., were different from fibrocytes or CAFs). Additionally, mutations detected in CTC^FAPα^ in M-CRC and L/M-PDAC and L-EOC implied neoplastic character, unlike CAFs.

**Fig. 6:**
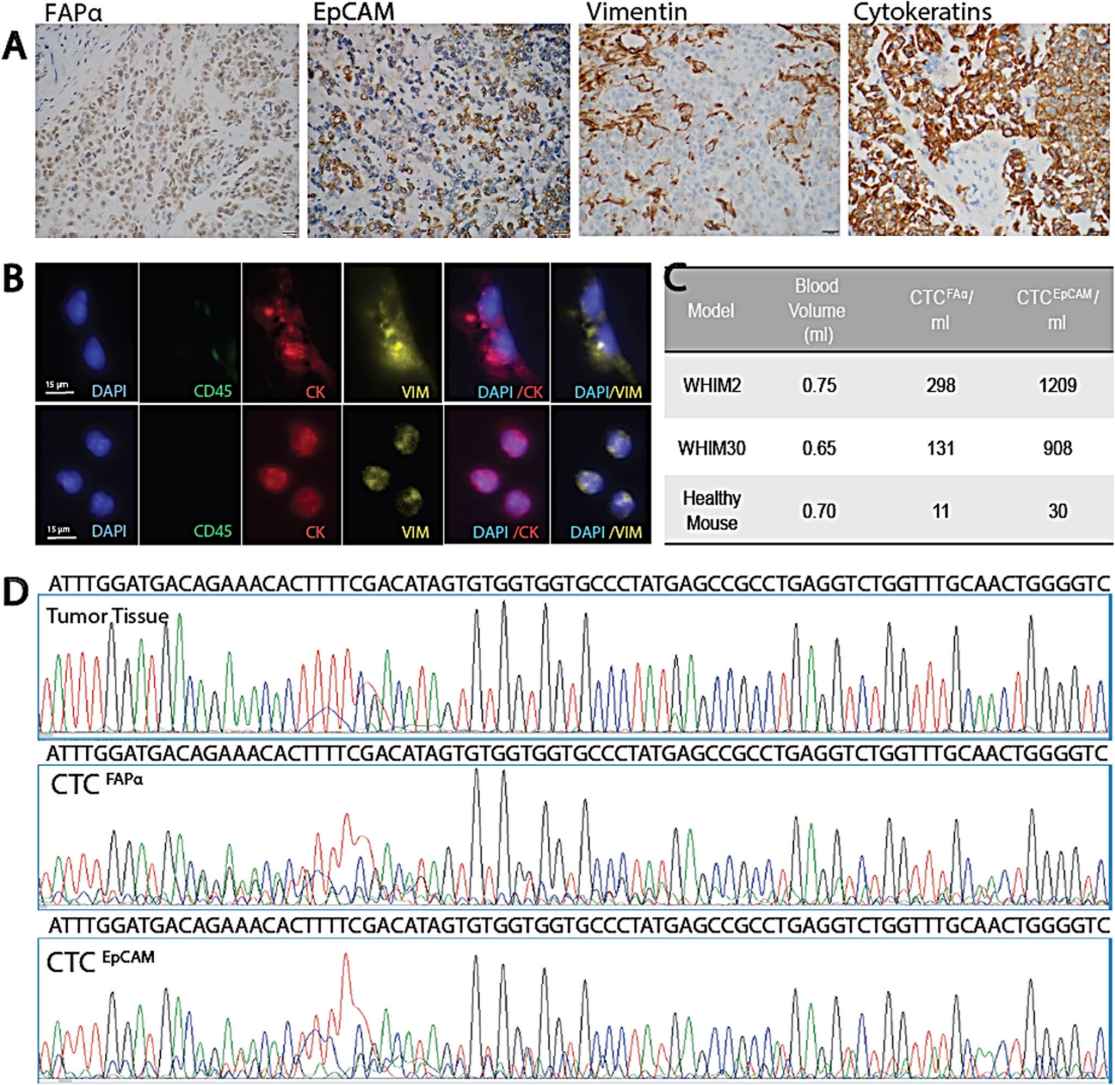
Phenotype, genotype, and CTCs from basal-like breast cancer PDX models. **a** IHC (400×) of tumor tissue in paraffin sections stained for FAPα, EpCAM, VIM, and pan-CK (scale bar = 20 µm). **b** Fluorescence microscope images of CTC^FAPα^ and CTC^EpCAM^ isolated from the blood via cardiac puncture (scale bar = 15 µm). **c** CTCs isolated from two PDX models and a healthy NSG control. **d** Sanger sequencing traces for amplicons generated from exon 6 *TP53* DNA isolated from tumor tissue, CTC^FAPα^, and CTC^EpCAM^ with primers designed for human sequence

We used PDX mouse models to more directly test whether isolated CTC^FAPα^ originated from human tumor or activated stroma (i.e., mouse stroma). We note that the anti-human mAbs used for isolation in this study will cross react with murine FAPα and EpCAM antigens; (human FAPα shares 90% AA identity with mouse FAPα, and human/mouse EpCAM share 82% aa sequence identity). CTC originating from mouse tissue and human tumor will be detected. We isolated CTCs from PDX models of basal-like breast cancer (Fig. 6a–c) and extracted gDNA from CTC subpopulations and tumor tissue. CTCs gDNA was subjected to WGA, PCR amplification with human-specific primers, and sequencing. The sequences were evaluated for homology to human and mouse gDNA of the same exon. The DNA from both CTC^EpCAM^ and CTC^FAPα^ subpopulations and tumor showed human sequence (Fig. 6d), suggesting CTC^FAPα^ did not originate from mouse stroma surrounding the tumor.

It is possible that CTC^FAPα^ originate from epithelial precursor cells or are a product of independent event in the epithelium, or could be precursor cells themselves with the ability to differentiate and clonally expand; these cells could represent a subpopulation of cancer cells undergoing EMT or mesenchymal–epithelial transition.^37^

## Discussion

The challenge associated with CTCs as biomarkers has been modest clinical sensitivity with the FDA-approved platform. The question arises: does the biology limit the CTC burden or is the analytical platform used for their isolation limiting? Indeed, many microfluidic technologies have shown higher clinical sensitivity/CTC test positivity compared to the FDA-approved test.^6, 38^ A challenge with CTC assays is that in many cases, only a single selection marker is used for isolation despite the phenotypically diverse microenvironment of the tumor. We addressed this issue by using a CTC dual selection strategy that employed discrete microfluidics designed to independently select two phenotypically distinct subpopulations; CTC^EpCAM^ and CTC^FAPα^, which represent epithelial and mesenchymal-like cancer cell phenotypes, respectively. Dual selection with the use of discrete microfluidics provided high CTC test positivity and specificity (Fig. 1h). The orthogonality of these two subpopulations was demonstrated through differential expression of *EpCAM* and *FAPα* mRNA and immunophenotyping with anti-EpCAM and anti-FAPα antibodies (Fig. 2, Supplementary Fig. S7A). For CRPC patients, *PSA* and *PSMA* mRNA expression in CTC^FAPα^ indicated that these cells originated from the prostate tumor (Supplementary Fig. S7B), which is not unprecedented as EpCAM-/PSMA+ prostate cancer CTCs have also been identified by others.^39^

FAPα as a new marker for CTC affinity selection was specific as only a few hematopoietic cells were co-isolated from blood of healthy donors and non-cancer patients. Although circulating fibroblasts (FAPα^+^/ α-SMA^+^/CK^−^/CD45^−^) were found in metastatic cancer patients (median = 4/7.5 ml) using filtration,^34^ these cells were not consistently isolated in our studies. These cells were CK−/CD45−/VIM+, and with this distinct phenotype, we could distinguish these cells from CTCs without compromising the integrity of the dual selection assay.

High purities of both CTC subpopulations allowed for bulk molecular analyses, obviating the need for single cell analysis. For example, a chemotherapy naïve L-EOC patient sample with high CTC^FAPα^ and CTC^EpCAM^ counts underwent WGA of gDNA and targeted-exome NGS; similar mutational profiles between the CTC subpopulations suggested a common origin. It is possible that unique mutation profiles in subclones of CTC^FAPα^ and/or CTC^EpCAM^ subpopulations were too infrequent to be detected by bulk analysis. Even with the purity achieved herein, detecting mutations by NGS in low frequency clones would incur requiring a more rigorous workflow including single CTC picking, WGA, NGS, and comparative analysis of single CTC mutations (consensus sequencing). Unfortunately, associated with this workflow would be amplification errors and low success rates associated with WGA.^40^ The costs and intense labor associated with these strategies would hinder clinical translation. Alternatively, PCR/LDR provides enhanced sensitivity for low frequency mutations, thereby providing translatable analysis of actionable and highly conserved mutations, such as *KRAS*.

*KRAS* mutational status in CTCs has been shown to have a high concordance with the primary tumor (~90%).^30^ Thus, in the absence of a primary tumor or anatomically inaccessible organs, decisions regarding treatment appropriateness could be made using CTCs.^30^ This is important as patients who harbor *KRAS* mutated genes derive minimal benefit from anti-EGFR mAb therapy. We detected *KRAS* mutations in CTC^FAPα^ and CTC^EpCAM^ but not always in both CTC subpopulations. For example, in two PDAC patients who underwent multiple rounds of chemotherapy, *KRAS* mutations were found in CTC^FAPα^ but not in CTC^EpCAM^ (Fig. 5). Thus, the testing of both subpopulations would be advisable to secure better concordance with the primary/metastatic sites and provide information for combination therapies. These differences in mutational status are not clear but may be a result of chemotherapy or reflect different cancer cells’ pre-chemotherapy *KRAS* dependence.^41^ Interestingly, M-EOC patients were found to have a 3-fold lower median CTC^EpCAM^ count following chemotherapy treatment compared to chemotherapy naïve patients, while CTC^FAPα^ median counts for these cohorts remained unchanged, potentially suggesting CTC^EpCAM^ were more sensitive to chemotherapy. Further studies should address whether CTC^FAPα^ are equipped with properties that enable chemoresistance.

Recently, the presence of both epithelial and quasi-mesenchymal subtypes of cancer cells was identified in PDAC^42^; selection strategies targeting EpCAM only may not fully recapitulate the primary/metastatic tumor and provide insufficient information for patients with a non-epithelial PDAC subtype. We longitudinally tracked CTC^FAPα^ and CTC^EpCAM^ in L/M-PDAC patients and observed that CTC^EpCAM^ burden alone was not indicative of disease status (Fig. 4); however, the ratio of CTC^FAPα^ to CTC^EpCAM^ better correlated with disease progression (Supplementary Fig. S5).

A dual selection strategy with orthogonal markers offered high test positivity for CTCs for a several cancers, even early stage disease. In addition, we demonstrated the ability to efficiently isolate CTCs from small blood volumes in PDX mouse models. The sinusoidal microfluidic chips provided high recovery and purity of CTCs for both localized and metastatic cancers to allow for “bulk” molecular profiling. Further, the use of discrete microfluidics for dual selection of CTCs of different phenotypes obviated the loss of subpopulation-specific distinctions in therapy response due to ensemble averaging, which would occur if mixed-monolayers of mAbs were poised within one microfluidic device.

Surveillance of both CTC subpopulations (epithelial and mesenchymal) can deliver more phenotype-specific insights into cancer progression and chemotherapy resistance, which cannot be discerned using other types of circulating markers, e.g., cell free DNA, because their origin cannot be associated with a certain tumor cell type.

## Methods

### Clinical samples

Healthy donors’ blood samples were obtained from the UNC Cancer Hospital Blood Bank. Blood from patients diagnosed with non-cancer or cancer were collected according to an approved UNC Institutional Review Board procedure. Written informed consent was obtained from all patients included in the study before enrollment. Peripheral blood samples were drawn by venipuncture into Vacuette^®^ containing EDTA (Greiner) tubes. Tables S2–S8 provide annotation data on the patients enrolled in this study. Supplementary Tables S14–S19 provide raw CTC enumeration data.

### Reagents and chemicals

COC (6013S-04) was purchased from TOPAS Advanced Polymers (Florence, KY). Chemicals and reagents used in these studies included Micro-90, reagent-grade isopropyl alcohol (IPA), phosphate-buffered saline pH = 7.4 (PBS), 2-(4-morpholino)-ethane sulfonic acid (MES), 7.5% bovine serum albumin (BSA), Triton X-100, paraformaldehyde solution (Sigma-Aldrich, St. Louis, MO), 1-ethyl-3-(3-dimethylaminopropyl)carbodiimide (EDC), N-hydroxysuccinimide (NHS) (Pierce, Rockford, IL), mouse anti-human EpCAM mAb (R&D Systems, clone#158210, Minneapolis, MN), mouse anti-human Fibroblast Activation Protein α (FAPα) mAb (R&D Systems, clone#427819), mouse monoclonal anti-Fc blocker IgG (R&D Systems,), DAPI, anti-CD45-FITC mAb (eBioscience, clone HI30), anti-CK 8 and 19 mAb (CK8/19-eFluor^®^615, clone#LP3K, BA17), anti-pan-CK-(AE1/AE3) eFluor^®^615 mAb (eBioscience, San Diego, CA), anti-human EpCAM-eFluor^®^660 mAb (eBioscience, clone#1B7), anti-human vimentin-Alexa Fluor^®^488 mAb (clone 280618 (R&D), and anti-human vimentin-APC mAb (R&D Systems, clone#280618). Nuclease-free water and microtubes (Ambion, Foster City, CA) were used for preparation and storage of all samples and reagents.

### Fabrication and assembly of the CTC microfluidic devices

Microfluidic devices used COC substrates that were hot embossed from a metal mold master. The chip design was a Z-configuration consisting of a 26.3 mm × 20.5 mm footprint with inlet and outlet channels (20.5 mm long, 400 µm wide, and 150 µm deep) connecting a series of 50-sinusoidal channels that in concert formed the CTC selection bed. Each sinusoidal channel was 30.6 mm long, 150 µm deep and 25 µm wide.

The surface area of the CTC selection bed was 596 mm^2^ (11 mm^2^/channel). The chip’s total volume was 9.4 µl (138 nl/channel) with a 2.5 µl volume for the inlet/outlet channels. Microfluidic devices and the planar substrates from which they were made were sonicated in 10% Micro-90 for 10 min, rinsed with IPA and DI water and dried at 70 °C. Devices and cover plates, both consisting of COC, were thermally fusion bonded between two glass plates in a convection oven at 131 °C for 30 min after which, they were UV/O_3_ activated for 15 min (22 mW/cm^2^ at 254 nm) in a home-built activation chamber equipped with a quartz, low-pressure Hg lamp. This activation protocol generated a functional scaffold of surface-confined carboxylic acids to which selection mAbs could be attached. Devices were modified using EDC–NHS chemistry (20 mg/ml EDC, 2 mg/ml NHS, in 100 mM MES, pH 4.8) followed by incubation with a solution of mAb (0.5 mg/ml; 150 mM PBS buffer, pH 7.4) overnight at 4 °C.

### Fluid dynamic simulations through sinusoidal channel architectures

The Chang–Hammer model^24^ (see Eq. 1) was used to investigate the dynamics of CTC affinity-selection as described elsewhere.^16^ Parameters for the simulation not provided by Chang–Hammer^24^ were: a 16-µm diameter CTC, mean EpCAM expression of 49,700 EpCAM molecules/cell, ^43^ a *k*_in_ of 2.5 × 10^4^ M^−1^ s^−1^ for antibody-EpCAM binding kinetics,^44^ and variable rolling distance.

### Isolation of CTC^FAPα^ and CTC^EpCAM^ via dual selection

Whole blood was processed using the dual selection strategy within 3 h following collection. Usually 2 ml of blood was infused into the microfluidic device yielding a linear velocity of 2 mm/s (25 µl/min). A post-isolation rinse was performed at 4 mm/s with 2 ml PBS/0.5% BSA. Affinity-bound cells were identified and enumerated via staining or impedance sensing.

### CTC staining and imaging

Cells were stained with anti-CD45-FITC mAbs (clone HI30; BioLegend, San Diego, CA), fixed with formaldehyde (2%), permeabilized with 0.1% Triton X-100, and stained with a mixture of CK 8, (clone C-46), 18 (clone DA/7), 19 (clone A53-B/A2), or pan-CK-eFluor^®^615 (clone C-11; BioLegend), anti-Vimentin-Cy5, and DAPI. In some cases, cells were stained with anti-EpCAM-Cy5 or FAPα via a secondary IgG mAb. CTC visualization/enumeration was performed using an inverted Olympus IX71 microscope (Center Valley, PA) equipped with a high resolution (1344 × 1024) CCD camera (Hamamatsu ORCA-03G) and a mercury arc lamp. Images were collected, background corrected, normalized, and analyzed using Metamorph software (Molecular Devices Inc.). In ImageJ images were converted to 8-bit type, a gray scale values of the signal were read from the line plots and the phenotypes were classified as no signal (−) (0–30 level), weak (+) (31–100 level), and strong (++) (101–256 level).

### Impedance detection of CTCs

Following CTC selection and bed washing, CTCs could be released from the capture surface of the sinusoidal channels with buffer consisting of 0.25% w/v trypsin in 25 mM TRIS/192 mM glycine buffer (pH 7.4). Released CTCs traversed through an impedance sensor and an electrical signal was recorded using in-house designed electronics. Impedance responses from CTCs were scored when the signal-to-noise ratio exceeded 3:1 using Matlab.

### RNA isolation and reverse transcription (RT)—quantitative PCR (RT-qPCR)

Cells were lysed and RNA was extracted from the lysate followed by RT performed using the Cell-to-Ct Kit (Life Technologies). A volume of2 μl of synthesized first strand cDNA was used for qPCR performed with a Universal SYBR green mix (BioRad) using a total reaction volume of 10 μl. RT-qPCR was performed using an Agilent HT7900 instrument (Applied Biosystems, Foster City, CA, USA). Primers were obtained from RealTimePrimers.com.

The qPCR steps consisted of 20 s at 95 °C and 40 cycles each for 3 s at 95 °C and 15 s at 58 °C and 15 s at 68 °C. Expression data were calculated using the comparative threshold cycle (*C*_t_) method. Glyceraldehyde-3-phosphate dehydrogenase (*GAPDH*) was used as the endogenous control. The *C*_t_ data for *GAPDH* was used to create Δ*C*_t_ values [Δ*C*_t_ = *C*_t_ (target gene)−*C*_t_ (*GAPDH*)]. Relative quantification values were calculated using the equation: 2^−ΔCt^.

### Genomic DNA isolation, whole genome amplification, and NGS

gDNA was extracted and purified using the Quick-gDNA™ MicroPrep kit (Zymo Research). WGA was performed using the Illustra Single Cell GenomiPhi DNA Amplification Kit (GE Healthcare) following manufacturer protocol. Samples were subjected to targeted-exome NGS on a Miseq^®^ using the TruSight™ Tumor 26 Sequencing Panel (Illumina).

### PCR/LDR assay

Cell lines of known *KRAS* genotype (HT29, wild-type and LS180—G12V) were secured from the Tissue Culture Facility at UNC. gDNA from the cell lines and CTCs was extracted using an Agencourt DNA isolation kit (Beckman–Coulter). PCR was performed with DNA in a total volume of 20 µl using *Taq* 2 × Master Mix (New England Biolabs, Ipswich, MA). PCR cocktails consisted of 2 µl of primers, 10 µl Taq 2× Master Mix, 6 µl nuclease free water and 2 µl gDNA. PCR was carried out in a thermal cycler (MJ Research Inc.) with the following steps: denaturation at 94 °C for 2.5 min followed by 40 cycles of denaturation at 94 °C for 15 s; annealing for 30 s at 58 °C and extension at 72 °C for 30 s. A final extension at 72 °C for 7 min was followed by a cooling step at 4 °C. *KRAS* primers were obtained from IDTDNA: forward primer 5′ AAC CTT ATG TGT GAC ATG TTC TAA TAT AGT CAC 3′ and reverse primer 5′ AAA ATG GTC AGA GAA ACC TTT ATC TGT ATC-3′. PCR products (290 bp) were electrophoresed at 8.3 V/cm in 1 × TBE using a 4% agarose gel with ethidium bromide (Lonza) staining. Amplicons were indexed against a DNA sizing ladder 50–766 bp (New England Biolabs). Images were collected using a Logic Gel imaging system (Eastman Kodak).

LDRs were carried out in a 20-µl volume with North9^o^ Ligase. The LDR cocktail contained discriminating and common primers 4 nM each, amplicons 0.6–1 ng (3–5 fmol), 40 units of DNA ligase and buffer. Thermocycling conditions were 94 °C for 1 min and 59 °C for 4 min that was repeated 20-times. Common primers for codon 34 and 35 were Cy5-labeled. Discriminating primers were design to produce ligated products with different sizes (Supplementary Table S13 **)**. LDR products were separated using a Beckman CQ CE system and sized against the appropriate ladder.

### Statistical analysis

Statistical analysis was conducted using a non-parametric *U*-test (Wilcoxon–Mann–Whitney test). For all analyses, *p* < 0.05 was considered statistically significant.

### Data and materials availability

The authors declare that all data supporting the findings of this study are available within the paper and its supplementary information files. Discussion of data contained within this study or its relevant findings can be addressed by the corresponding author upon reasonable request.

## Electronic supplementary material

Supplemental material

## Electronic supplementary material

**Supplementary Information** accompanies the paper on the *npj Precision Oncology* website (doi:10.1038/s41698-017-0028-8).
