# Supplementary material for: Discrete microfluidics for the isolation of circulating tumor cell subpopulations targeting fibroblast activation protein alpha and epithelial cell adhesion molecule
Source: NPJ Precis Oncol. 2017 Jul 25;1:24. doi: 10.1038/s41698-017-0028-8 (PMC5871807; doi:10.1038/s41698-017-0028-8)
Supplement: Supplementary file 1 — Supplemental material [file 41698_2017_28_MOESM1_ESM.docx]

Supplemental Information

**Discrete Microfluidics for the Isolation of Circulating Tumor Cell Subpopulations Targeting Fibroblast Activation Protein alpha and Epithelial Cell Adhesion Molecule**

Małgorzata A. Witek,^1,2,3^ Rachel D. Aufforth,^4^ Hong Wang,^3^ Joyce W. Kamande,^3^ Joshua M. Jackson,^1,2^  Swathi R. Pullagurla, ^1,2^ Mateusz L. Hupert,^3,5^ Jerry Usary,^6,8^ Weiya Z. Wysham,^7,8^ Dawud Hilliard,^8,9^ Stephanie Montgomery,^9,10^ Victoria Bae-Jump,^7,8^ Lisa A. Carey,^8,11^ Paola A. Gehrig, ^7,8^

Matthew I. Milowsky,^8,11^ Charles M. Perou,^6,8,11^ John T. Soper, ^7,8^ Young E. Whang, ^8,10,11^

Jen Jen Yeh,^4,8,11,12*^ George Martin,^13*^ and Steven A. Soper ^1,2,5,14,15,16*^

^1^ Department of Chemistry, The University of Kansas, Lawrence, KS 66047

^2^ Center of Biomodular Multiscale Systems for Precision Medicine, The University of Kansas, Lawrence, KS 66047

^3^ Department of Biomedical Engineering, The University of North Carolina, Chapel Hill, NC 27599

^4^ Department of Surgery, Division of Surgical Oncology and Endocrine Surgery, UNC-Chapel Hill, NC 27599

^5^ BioFluidica, Inc., c/o Carolina Kick-Start, 321 Bondurant Hall, Chapel Hill, NC27599

^6^ Department of Genetics, The University of North Carolina, Chapel Hill, NC 27599

^7^ Division of Gynecologic Oncology, Department of Obstetrics and Gynecology, UNC-Chapel Hill, NC 27599

^8^ Lineberger Comprehensive Cancer Center, The University of North Carolina, Chapel Hill, NC 27599

^9^ Animal Histopathology Core, The University of North Carolina, Chapel Hill, NC 27599

^10^ Department of Pathology and Laboratory Medicine, The University of North Carolina, Chapel Hill, NC 27599

^11^ Department of Medicine, Division of Hematology and Oncology, The University of North Carolina, Chapel Hill, NC 27599

^12^ Department of Pharmacology, The University of North Carolina, Chapel Hill, NC 27599

^13^ Roche, Pleasanton, CA 94588

^14^ BioEngineering Program, The University of Kansas, Lawrence, KS 66047

^15^ Department of Mechanical Engineering, The University of Kansas, Lawrence, KS 66047

^16^ Ulsan National Institute of Science and Technology, Ulsan, Republic of Korea

* Authors to whom correspondence should be addressed: [jen_jen_yeh@med.unc.edu](https://outlook.unc.edu/owa/redir.aspx?SURL=sl42EfT8NP6vSPT7k6T8ClrNryJb78JtRAm4x-C2rRWsdPcFsdjSCG0AYQBpAGwAdABvADoAagBlAG4AXwBqAGUAbgBfAHkAZQBoAEAAbQBlAGQALgB1AG4AYwAuAGUAZAB1AA..&URL=mailto%3ajen_jen_yeh%40med.unc.edu) (J.J.Y.); [george.martin@roche.com](mailto:george.martin@roche.com) (G.M.); [ssoper@ku.edu](mailto:ssoper@ku.edu) (S.A.S.).

**Table of Contents**

**Experimental Methods page #**

Study design …………………………………………………………………………………………………………………………………. 3

Cell culture and flow cytometry of model cell lines (Hs578T and SKBR3) …………………………………………………………… .3

Self-referencing method.……………………………………………………………………………………………………………………. 3

Patient Derived Xenograft (PDX) models ………………………………………………………………………………………………….4

Tumor tissue staining ……………………………………………………………………………………………………………………… .4

CTC lysis and molecular profiling on CRPC samples ……………………………………………………………………………………5

**Supplementary Figures**

Figure S1. Expression of FAPα and EpCAM in cancer cell lines …….…………………………………………………………………6

Figure S2. Characterization of model cell lines ….………………………………………………………………………………..……….7

Figure S3. Cell counting via impedance ……………………………………………………………………………………………………8

Figure S4. Summary of enumeration of CTCs in EOC patients ….……………………………………………………………….…….9

Figure S5. Summary of longitudinal tracking data in L/M-PDAC patients ……………………………………………………………..10

Figure S6. Relative expressions of mRNA for selected genes evaluated for Hs578T and SKBR3 cell lines harvested from the culture flask and affinity isolated on a microfluidic chip ………………………………………………………………………………….11

Figure S7. Summary of the average relative expressions of mRNA for selected genes ..…………………………………………...12

**Supplementary Tables**

Table S1**.** Variations in CTC counts when the order of the FAPα and EpCAM devices was changed …………………………….13

Table S2. CTC enumeration in healthy donors …………………………………………………………………………………………..13

Table S3. CTC enumeration in non-cancer patients ………………………………………………………………………………….…13

Table S4. Metastatic breast cancer patients enrolled in the study ……………………………………………………………..………14

Table S5. PDAC patients enrolled in the study ……………………………………………………………………………………..……15

Table S6. PDAC patients’ disease progression and treatment …………………………………………………………...…………….16

Table S7. Epithelial ovarian cancer (EOC) patients enrolled in the study ………………………………………………….………...17

Table S8. CRPC patients enrolled in the study …………………………………………………………………………………………..18

Table S9. Comparison of CTC numbers secured via phenotyping and impedance sensor ………………………………………...18

Table S10. CTC enumeration summary from all patients ……………………………………………………………………..………..19

Table S11. Summary of pairwise statistical analyses of CTC counts …………………………………………………………..………20

Table S12. Mutations detected in NGS for CTCs subpopulations isolated from an ovarian cancer patient ……………………...21

Table S13. Primers sequences used in LDR …………………………………………………………………………………………....22

Table S14. CTC^FAPα^ and CTC^EpCAM^ isolated from the blood of metastatic breast ductal carcinoma patients ..………………..…23

Table S15. CTC^FAPα^ and CTC^EpCAM^ isolated from the blood of M-PDAC patients …………….…….............................................24

Table S16. CTC^FAPα^ and CTC^EpCAM^ isolated from blood of L-PDAC patients ………………………………………………………..25

Table S17. CTC^FAPα^ and CTC^EpCAM^ isolated from the blood of colorectal cancer patients ……………………… …………………26

Table S18. CTC^FAPα^ and CTC^EpCAM^ isolated from the blood of castration resistant prostate cancer patients …………..………..26

Table S19. CTC^FAPα^ and CTC^EpCAM^ isolated from the blood of epithelial ovarian cancer patients ……………………..………….27

**References** ………………………………………………………………………………………………………………………………….28

**Experimental Methods**

**Study design.** This work was instigated based upon the hypothesis: *FAPα, which is expressed on cells comprising the tumor microenvironment, can be used as an additional marker for selecting a phenotypically distinct CTC subpopulation with respect to a CTC subpopulation that expresses EpCAM*. To test this hypothesis, we conducted a prospective trial with patients diagnosed with five different malignancies: CRPC, CRC, BC, PDAC, and EOC. We evaluated the following: Correlation of CTC numbers from each subpopulation with (i) disease burden, (ii) surveillance of recurrence, and (iii) ability to monitor treatment response. Longitudinal studies in PDAC patients was possible during a post-surgical follow-up. The individuals analyzing samples knew from what patient group blood was collected, but were blinded to the distinction between patients’ type of treatment, disease stage, type of tumor, benign condition, etc. After completion of the study, patients’ cancer characteristics (*i.e.,* histologic/pathologic type, stage/grade, disease recurrence via CT scans), history and prior treatments were obtained from the study coordinator and cohorts were established.

**Cell culture and flow cytometry of model cell lines (Hs578T and SKBR3).** The Hs578T cell line was cultivated in 1× MEM, 1× NEAA and 10% FBS. SKBR3 cells were grown in 1× McCoy Medium and 10% FBS. Cultures were incubated at 37^o^ C with 5% CO_2_. Cells were released from the flask surface with TrypLE^™^ reagent (Gibco). Flow cytometry was performed with a CyAn flow cytometer (Beckman-Coulter) equipped with a 25 mW 488 nm laser. Ten thousand events were counted for all samples. Data acquisition and analysis was performed using Summit software (Dako, Carpineteria, CA). Prior to staining, the cells’ surface were blocked with human IgG and incubated at 4º C. The following samples were prepared for analysis: (i) Unstained cells serving as an autofluorescence control; (ii) cells incubated with propidium iodide (PI); (iii) cells stained with 10 µl of 0.1 mg/ml isotype control, IgG_1_-FITC mAbs (R&D Systems, Minneapolis, MN) for Hs578T and IgG_2_ for SKBR3; (iv) cells incubated with goat anti-mouse secondary IgG-FITC; (v) cells stained with mouse anti-human EpCAM-FITC Ab and incubated in the dark at 4º C for 30 min; and (vi) cells stained with 10 µl of 0.1 mg/ml CD4-FITC mAb (BD Biosciences, Franklin Lakes, NJ) and incubated in the dark at 4º C for 30 min. Cells were washed three times with 1 ml of cold PBS/0.5% BSA. PI staining (0.5 µg/ml) was performed on all of the samples for determining cell viability.

**Self-referencing method.** Quantifying the number of selected cells recovered is typically accomplished using seeding experiments in which a known number of target cells are introduced into a suspension. Unfortunately, this technique does not allow one to determine recovery of target cells present in clinical samples because the cell frequency is unknown. We developed a “self-referencing” method, where prior knowledge of the number of target cells is not required. The self-referencing method uses multiple cell selection devices connected in series. The number of cells isolated in the first device divided by the total cell count from all devices in the series quantifies recovery. An error in the quantification is low (<7%) at high device recovery (70-100%), requiring only two devices in series; the error can be minimized for this measurement scheme with three devices in series when the recovery is <60%. Thus, the self-referencing method can accurately measure a device’s recovery from samples and with low standard deviations. Using this method, we calculated the recovery from following equation:

$$\frac{CTC_{chip 1}}{CTC_{total}}=\frac{CTC_{chip 1}}{CTC_{chip 1}+CTC_{chip 2}+\ldots CTC_{chip N}}$$

**Patient Derived Xenograft (PDX) models.** All animal procedures were done under protocols approved by the University of North Carolina Institutional Animal Care and Use Committee. PDX tumors were obtained from the Washington University School of Medicine in St. Louis (http://digitalcommons.wustl.edu/hamlet/). Two PDX models (WHIM2 and WHIM30) corresponding to the basal-like breast cancer subtype were used. Tumors were established in NOD *scid* gamma (NSG) (NOD.Cg-*Prkdc^scid^ Il2rg^tm1Wjl^*/SzJ) mice. The mice were briefly anesthetized with 2% isoflurane and ~2 million tumor cells in 50% Matrigel and 50% Hanks Balanced Salt Solution were subcutaneously injected into the fat pad of the fourth (inguinal) mammary gland. Mice were euthanized and the PDX tumors were removed when their size reached ~12 mm × 12 mm. Mouse terminal bleeds were performed via cardiac puncture. Blood was collected into 1 ml EDTA tubes and processed within 2 h of collection. Both CTC^FAPα^ and CTC^EpCAM^ were collected using the sinusoidal microfluidic chip. Genomic DNA (gDNA) was isolated and whole-genome amplification (WGA) was performed using the Illustra Single Cell GenomiPhi DNA Amplification Kit (GE Healthcare) according to the manufacturer’s protocol. Exon 6 of the *TP53* was amplified via PCR with the forward primer – 5’CCTCTGATTCCTCACTGATTGCTCTTA3’; and a reverse primer with sequence – 5’GGCCACTGACAACCACCCTTAAC3’. Twenty ng of template per PCR was used with the following thermal cycling steps: Denaturation at 94˚C for 2.5 min followed by 40 cycles of denaturation at 94˚ C for 15 s; annealing for 30 s at 58˚ C and extension at 72˚ C for 30 s. A final extension at 72˚ C for 7 min was followed by a cooling step at 4˚ C. PCR products (199 bp) were electrophoresed at 8.3 V/cm in 1X TBE (Tris–boric acid/EDTA, Bio-Rad Laboratories) on a 4% agarose gel with ethidium bromide (Lonza) staining. Amplicons were excised from the gel, purified, treated with ExoSAP-IT reagent (Affymetrix) and Sanger sequencing performed.

**Tumor tissue staining.** Tissues were fixed in 1× Zn fixative (formalin free, BD Pharmingen™ IHC Zinc Fixative) for 48 h at room temperature followed by 24-48 h storage in 70% ethanol. Three mm thick blocks obtained from PDX primary tumor were embedded in paraffin. Four µm thick sections were cut from the block and placed on slides with the slides incubated at 70º C for 25 min. Staining was performed for immunohistochemical analysis using: (1) Monoclonal pan-CK antibody (MA5-13203, Life Technologies, 1:100, no antigen retrieval); (ii) monoclonal anti-CK19 antibody (MA512319, Life Technologies, 1:500, no antigen retrieval); (iii)  monoclonal anti-EpCAM antibody (clone#158210, R&D, 1:100, antigen retrieval using Ventana’s CC1 (pH 8.5), for 16 min at 100º C,); and (iv) monoclonal anti-Vimentin antibody (ab92547, Abcam, 1:250, antigen retrieval using Ventana’s CC1 (pH 8.5) for 64 min at 100º C). Antibody dilutions were prepared using the Dako ARK Kit (Animal Research Kit, K3954). The dilutions were incubated in a biotinylated reagent for 15 min at room temperature, followed by addition of a blocking solution and incubation for 5 min at room temperature. The samples were given a hydrogen peroxide block for 8 min at room temperature and then, incubated in the primary Ab for 1 h at room temperature, followed by the secondary Ab (Dako Ark Kit Streptavidin HRP solution) for 16 - 32 min at 35º C. The samples were treated with DAB, Hematoxylin II for 8 min, and then Bluing Reagent for 4 min followed by monoclonal anti-FAPα antibody (427819, R&D, 1:200, antigen retrieval using Ventana’s CC1 (pH 8.5), 72 min at 100º C). The slides were incubated in avidin and biotin (Ventana’s A/B Block, 760-050) for 8 min each, followed by a hydrogen peroxide block for 8 min at room temperature and then, incubated in the primary Ab for 6 h at room temperature. The slides were incubated in the secondary Ab (Anti-IgG1 + IgG2a + IgG3 antibody, Abcam, ab133469, 1:500) for 1 h, followed by a tertiary Ab (Ventana Omap OmniMap anti Rabbit HRP, 760-4311, Ready to Use) for 32 min at room temperature. The samples were treated with DAB, Hematoxylin II for 8 min and then, Bluing Reagent for 4 min. All slides were visualized using Ventana’s Discovery Ultra Automated IHC staining system.

**CTC lysis and molecular profiling on CRPC samples.** On-chip CTC lysis and total RNA extraction were performed using a protocol adapted from RNeasy micro kit (Catalog no. 74004, Qiagen). Briefly, following CTC selection, the chips were flushed with 1 ml of cold 1× PBS at 60 µl/min. Three-hundred µl of RLT buffer was flowed through the CTC microfluidic chip at a flow rate of 60 µl/min. Twenty ng of carrier RNA was added to the eluent and RTL buffer to obtain 350 μl as the final volume of the lysate. Then, 350 μl of 70% ethanol was added to the lysate and mixed by pipetting. The sample was transferred to an RNeasy MinElute spin column placed in a 2 ml collection tube and centrifuged for 30 s at 10,000× g. The column was washed with 350 μl Buffer RW1 (centrifuged at 10,000× for 30 s). Eighty μl of DNase I was added to the RNeasy MinElute spin column membrane and incubated at room temperature for 15 min. RNeasy MinElute spin column was then washed with 350 μl Buffer RW1 and 500 μl Buffer RPE (10,000x for 30 s). After that, the column was washed using 500 μl of 80% ethanol and dried by centrifuging 5 min at 18,000×. Finally, RNA was eluted using 14 μl RNase-free water (1 min centrifuge at 18,000× g). Reverse transcription was performed using SuperScript III reverse transcriptase (Catalog no. 18080, Invitrogen) and 8 µl of the extracted RNA. PCR was performed using primers targeting *GAPDH* (housekeeping control), *PSMA, PSA, AR, EpCAM, FAPα1*, and *FAPα2*.

**SUPPLEMENTARY FIGURES**

**
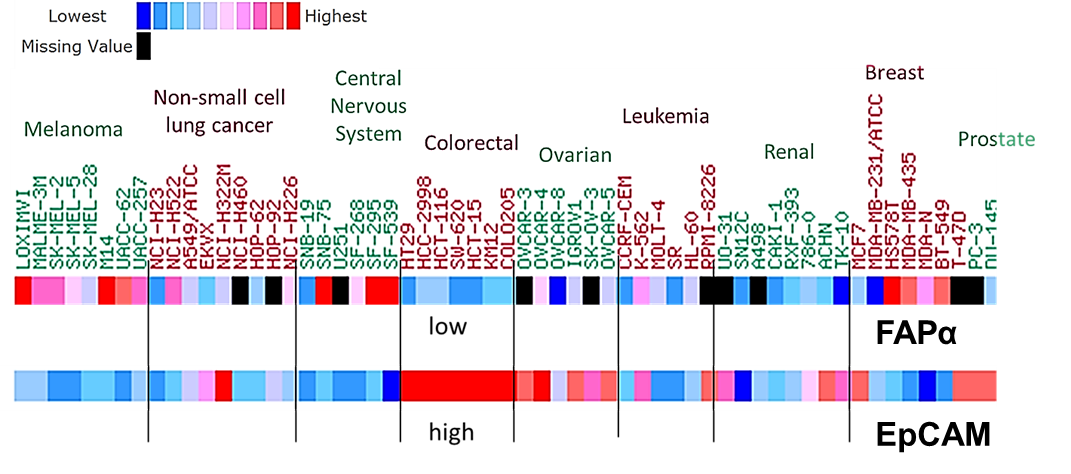
**

**Figure S1.** Expression of FAPα and EpCAM in various cancer cell lines. Data was secured from the NIH cancer genome atlas project as of 2013 (<http://cgap.nci.nih.gov>). Similar data can be now secured at <http://www.ebi.ac.uk/>.


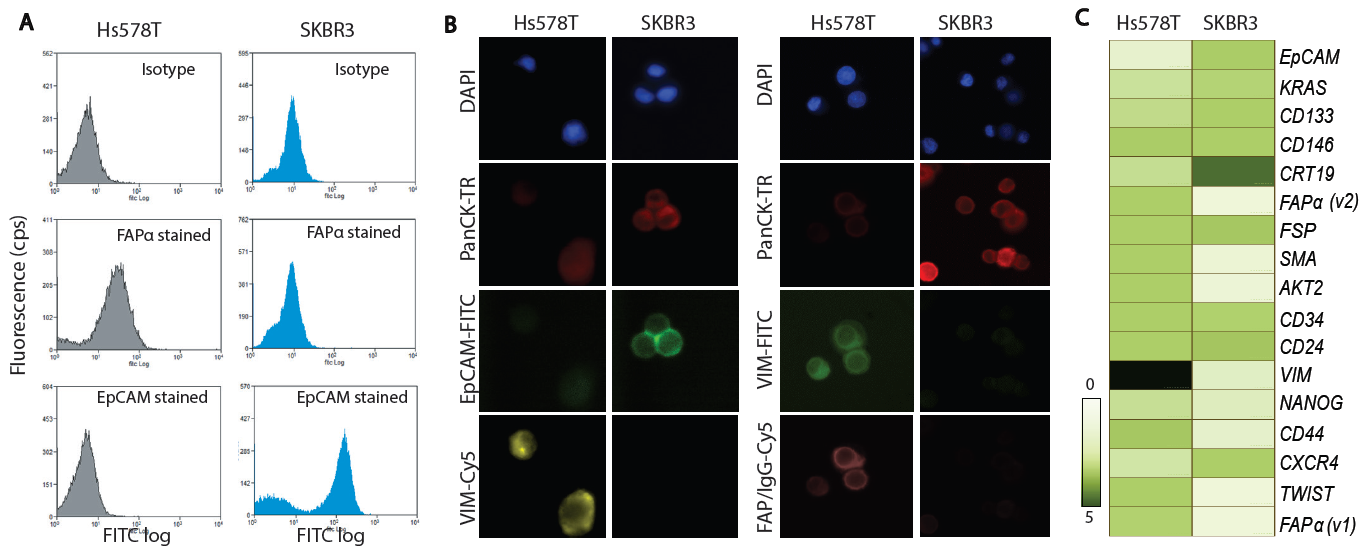


**Figure S2.** Characterization of model cell lines. **(A)** Multi-parameter flow cytometry (MFC), **(B)** immunostaining, and **(C)** RT-qPCR. **(A)** Histograms show MFC results for stained Hs578T and SKBR3 cells with the IgG isotype, FAPα via a secondary mAb, and anti-EpCAM mAb. **(B)** Immunostaining with epithelial and mesenchymal markers showing images of breast cancer cell lines stained with DAPI, Pan-CK, anti-EpCAM, and anti-Vim mAbs. Hs578T cell line (Claudin-like) is derived from a mammary gland of a carcinosarcoma. These cells showed mesenchymal and luminal morphology. SKBR3 cell line was derived from a metastatic site of a mammary gland adenocarcinoma. mAbs were labeled with Cy5, FITC, or Texas Red (TR). **(C)** RT-qPCR mRNA expression profiles for Hs578T and SKBR3 cell lines relative to *GAPDH* expression.

Results indicated that Hs578T cells were EpCAM-, with the FAPα expression 6-fold greater than the IgG control (**Fig. S2A**). The SKBR3 cell line showed high expression of EpCAM (15-fold IgG control) and no expression of FAPα. Hs578T weakly expressed pan-CK and strongly expressed vimentin (VIM), while SKBR3 cells demonstrated strong pan-CK and no VIM expression (**Fig. S2B**). RT-qPCR revealed high expression of *EpCAM* and *CRT19* in the SKBR3 cell line, but not in the Hs578T cell line (**Fig. S2C**). As expected, SKBR3 *VIM* mRNA expression was low.

**
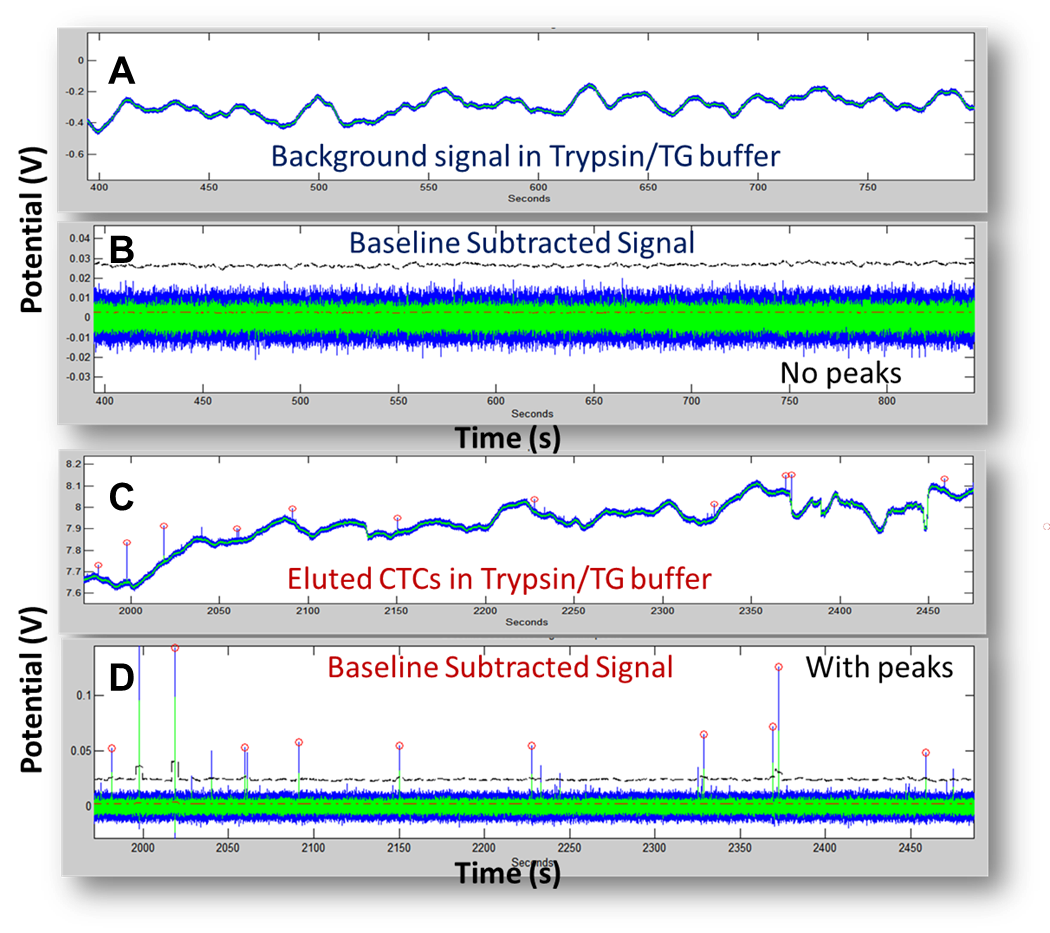
**

**Figure S3.** Cell counting via impedance detection. (A) Raw and (B) baseline subtracted signals for the enzyme/buffer system before cells are eluted, and (C) raw and (D) baseline subtracted signals for CTCs released from the sinusoidal CTC isolation device. Peaks observed in the traces in (C) and (D) correspond to single CTCs. Data analysis was performed with Matlab, and peak events were classified as CTCs when the signal magnitude was higher than 10x SD. The sensor operated at 40 kHz frequency; the flow rate was 25 µl/min; and the data collection frequency was 3 kHz. The Pt electrodes (75 µm) were separated by 50 μm producing a cell constant, K, equal to 0.01 μm^−1^ (defined as the ratio of electrode gap to the electrode area). *K* was scaled to specifically detect CTCs due to their larger size with respect to leukocytes or erythrocytes. As shown in our previous work, the conductivity sensor with K = 0.01 μm^−1^ provided near 100% CTC counting and does not transduce signals from leukocytes or erythrocytes due to their smaller size.[^1^](#_ENREF_1)^,^[^2^](#_ENREF_2)


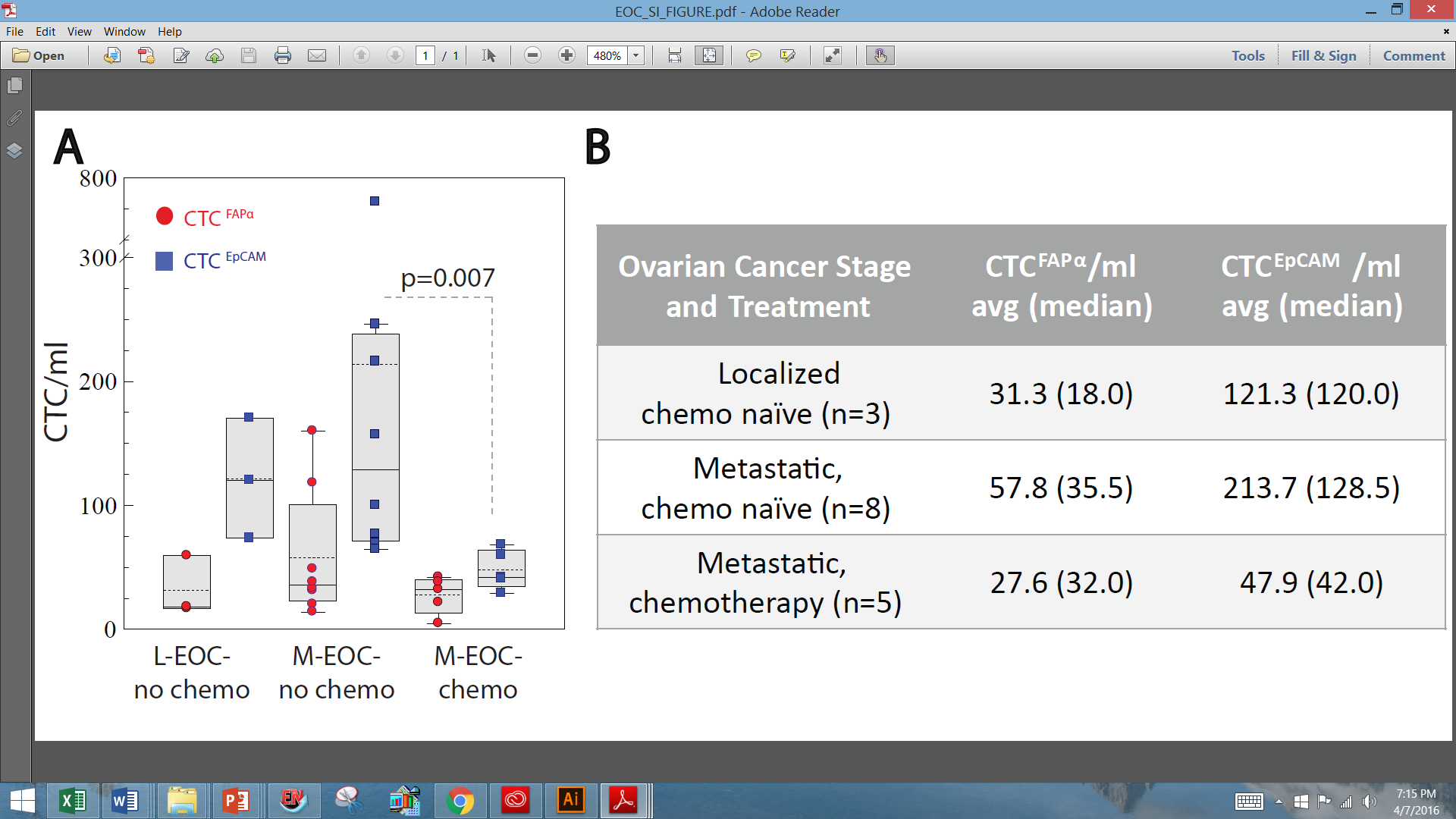


**Figure S4.** Enumeration data for CTCs in EOC patients**.** **(A)** Box plot for CTC^FAPα^ and CTC^EpCAM^ isolated from the blood of EOC patients: Non-metastatic chemo naïve (L-EOC-no chemo); metastatic chemo naïve (M-EOC-no chemo); and metastatic with chemotherapy treatment (M-EOC-chemo). **(B)** Tabulated average and median CTC enumeration data. The solid lines in the box plots represent the median and the dotted line is the mean for the data shown.

**
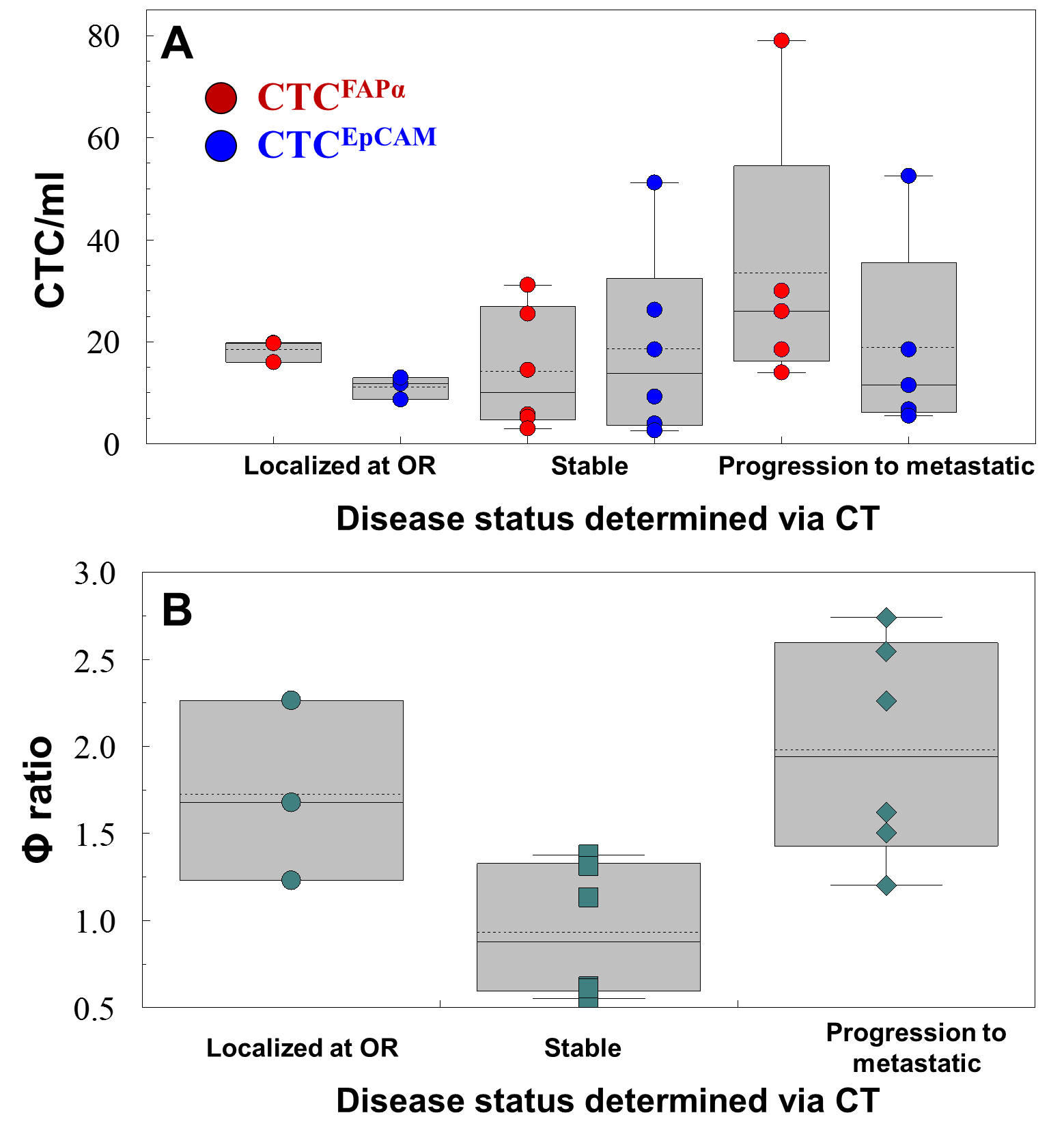
**

**Figure S5.** Summary of longitudinal tracking data in L/M-PDAC patients. (**A**) Box plot for CTC^FAPα^ and CTC^EpCAM^ counts isolated from the blood of L/M-PDAC patients, and (B) CTC^FAPα^/CTC^EpCAM^ ratio (*i.e*., ϕ ratio) calculated for the same patients at different stages of disease, as determined by CT imaging. Box plots represent upper and lower quartiles, dashed line is average and solid line is median. Localized at OR represents localized disease at time of surgical resection of tumor.


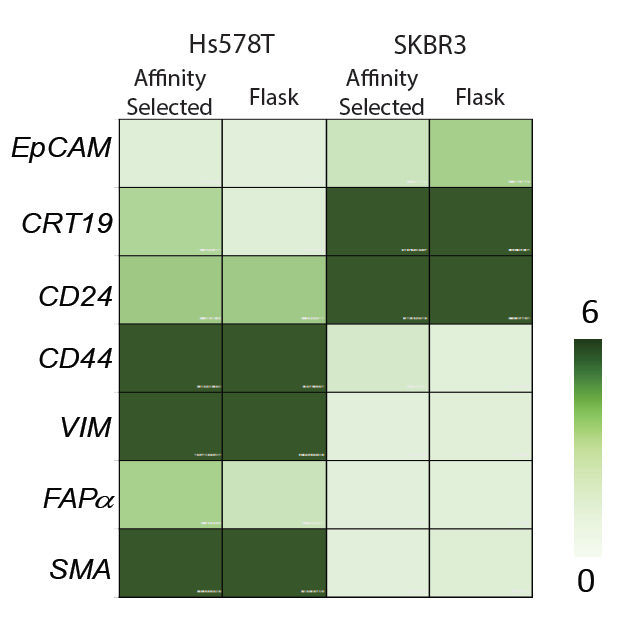


**Figure S6.** Relative expressions of mRNA for selected genes evaluated for Hs578T and SKBR3 cell lines for cells harvested from the culture flask and cells affinity isolated using the sinusoidal microfluidic chip. Approximately 200 cells were used in both experiments. Relative mRNA expression levels were calculated by the comparative CT method using GAPDH as an endogenous control.


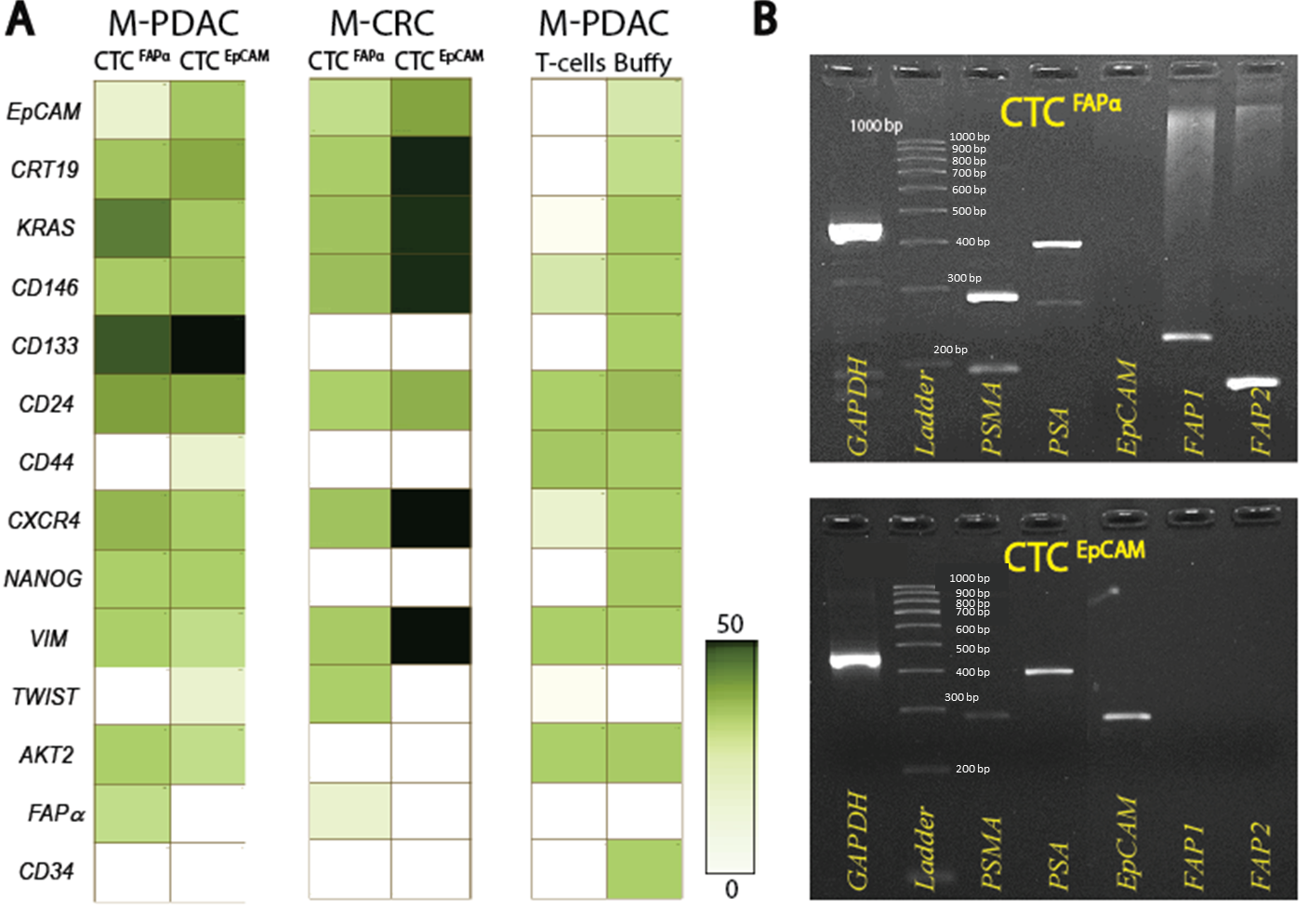


**Figure S7.** Average relative expressions of mRNA for selected genes from CTC^FAPα^ and CTC^EpCAM^. **(A)** mRNA expression heat plots for 5 M-PDAC, 2 M-CRC, T-Cells, and M-PDAC-buffy coat. Relative mRNA expression levels were calculated by the comparative CT method using GAPDH as an endogenous control. The average results (2^ΔCt^) obtained from RT-qPCR for all samples are summarized. Variations of *GAPDH* between samples were not evaluated. **(B)** Agarose gel fluorescence images for gene amplification after reverse transcription of mRNA taken from CTC^FAPα^ and CTC^EpCAM^ affinity selected from a CRPC patient. The samples shown here were derived from the same experiment and processed in parallel.

**SUPPLEMENTARY TABLES**

**Table S1.** Variations in CTC counts when the order of the FAPα and EpCAM devices was changed.

| Order  1^st^ chip→2^nd^ chip | CRC #116 | | PDAC#46 | | PDAC pt #66 | |
| --- | --- | --- | --- | --- | --- | --- |
|  | CTC^FAPα^ /ml | CTC^EpCAM/^ml | CTC^FAPα^ /ml | CTC^EpCAM/^ml | CTC^FAPα^ /ml | CTC^EpCAM/^ml |
| FAPα → EpCAM | 16 | 14 | 17.0 | 20.5 | 99.3 | 55 |
| EpCAM → FAPα | 11 | 12 | 12.0 | 32.0 | 58.7 | 50 |
| % RSD* | 26.2 | 10.9 | 24.4 | 30.9 | 36.3 | 6.7 |

*%RSD=(SD/Avg)x100

**Table S2.** CTC enumeration in healthy donors. Blood samples from 11 healthy donors were collected and between 1 and 5 ml of blood was processed using the dual selection assay.

| **ID** | **Clinical Sample** | **Analysis date** | **Blood volume processed (ml)** | **CTC^FAPα^ /ml (WBC/ml)** | **CTC^EpCAM^ /ml (WBC/ml)** |
| --- | --- | --- | --- | --- | --- |
| HD1 | Healthy Donor | 09/05/12 | 1.0 | 1 (3) | 0 (1) |
| HD2 | Healthy Donor | 09/05/12 | 2.0 | 0.5 (1.5) | 0 (0.5) |
| HD4 | Healthy Donor | 03/02/12 | 1.0 | 0 (3) | 0 (1) |
| HD5 | Healthy Donor | 04/12/13 | 2.0 | 0 (2) | 0.5 (0.5) |
| HD3 | Healthy Donor | 02/05/14 | 1.0 | 0 (3) | 0 (1) |
| HW16 | Healthy Donor | 07/05/12 | 2.5 | - | 0.4 (4.8) |
| HW1 | Healthy Donor | 07/12/12 | 2.5 | - | 0 (2) |
| HW2 | Healthy Donor | 07/12/12 | 2.5 | - | 0 (4) |
| HW3 | Healthy Donor | 08/16/12 | 5.0 | - | 0 (2.2) |
| HW4 | Healthy Donor | 08/30/12 | 1.5 | - | 0 (8.0) |
| HW5 | Healthy Donor | 08/30/12 | 1.5 | - | 0 (5.3) |

**Table S3.** CTC enumeration in non-cancer patients. Six patients with non-cancer diseases were enrolled in the study. Two ml of blood was processed using the dual selection strategy.

| **Patient ID** | **Diagnosis** | **Surgery date** | **Blood volume processed (ml)** | **CTC^FAPα^ /ml (WBC/ml)** | **CTC^EpCAM^ /ml (WBC/ml)** |
| --- | --- | --- | --- | --- | --- |
| 44 | Benign pancreas | 09/06/12 | 2 | 0 (0.5) | 0.5 (2) |
| 69 | Goiter | 12/11/12 | 2 | 1.5 (0) | 3.5 (0) |
| 71 | benign pancreas | 12/13/12 | 2 | 4.5 (2) | 4 (1) |
| 75 | adrenal mass | 12/19/12 | 2 | 1.5 (1) | 1 (0) |
| 81 | pancreatitis | 03/27/13 | 2 | 3.5 (0) | 4 (1) |
| 85 | benign pancreas | 03/22/13 | 2 | 0 (2) | 2.5 (2) |

**Table S4.** Metastatic breast cancer patients enrolled in the study.

| **Pt ID** | **Age** | **Race** | **Date of primary diagnosis** | **Date of metastatic diagnosis** | **Metastatic sites** | **Receptor status** | | | **Therapy at time of blood draw** | | | |
| --- | --- | --- | --- | --- | --- | --- | --- | --- | --- | --- | --- | --- |
|  |  |  |  |  |  | **Primary** | **Metastatic** | **Endocrine** | | **Chemo-**  **therapy** | **Comments** |  |
| #01 | 70 | C | 1981 | Dec 2008 | sternum, lungs, mediastinal lymph nodes | unknown | ER+/PR-/HER2- | Yes | | No | biologic |  |
| #02 | 69 | C | first presented with stage IV disease, Nov 2008 | | left axilla, breast, liver, bone, lung | unknown | ER+/PR+/  HER2+ | yes | | No |  |  |
| #03 | 65 | C | 2007 | Jun 2013 | lung, liver, bone | ER-/PR-/  HER2- | ER-/PR-/HER2- | No | | Yes |  |  |
| #04 | 62 | C | 2007 | Oct 2009 | liver, skin | ER+/PR+/  HER2+ | ER+/PR+/  HER2- | Yes | | No |  |  |
| #05 | 51 | C | 2010 | May 2012 | right axilla, mediastinum, left adrenal | ER+/PR-/  HER2- | ER-/PR-/HER2- | No | | yes | PD-L1 clinical trial |  |
| #06 | 56 | C | 2003 | Oct 2009 | bone, liver | ER+/HER2- | ER+/PR+/  HER2- | Yes | | No |  |  |
| #07 | 46 | C | first presented with stage IV disease, Jan 2013 | | bone, sacrum, lungs, brain | unknown | ER+/PR+/  HER2- | yes | | No |  |  |
| #08 | 36 | C | 2006 | July 2007 | left submandibular LN and mediastinum, right parietal | ER-/PR-/  HER2- | ER-/PR-/  HER2- | No | | No |  |  |
| #09 | 58 | AA | 2004 | Feb 2013 | bone, lung | ER+/PR+/  HER2- | ER+/PR+/  HER2+ | Yes | | No |  |  |
| #10 | 61 | C | 2000 | Aug 2008 | lung | ER+/PR+/  HER2- | ER+/PR+/  HER2- | No | | Yes |  |  |

**Table S5.** PDAC patients enrolled in the study.

| **Patient ID** | **Cancer stage at enrollment** | **Surgery** | **Status at Last Follow up** |
| --- | --- | --- | --- |
| 2 | Metastatic | N | Deceased |
| 24 | Metastatic | N | Deceased |
| 25 | Metastatic | N | Deceased |
| 40 | Metastatic | N | Deceased |
| 41 | Metastatic | N | Deceased |
| 42 | Metastatic | N | Deceased |
| 45 | local resectable | Y | Deceased |
| 46 | local resectable | Y | Alive, disease free |
| 47 | Metastatic | N | Deceased |
| 48 | local, resectable | Y | Deceased |
| 66 | local resectable | Y | Deceased |
| 67 | locally advanced unresectable | N | Deceased |
| 68 | Metastatic | N | Deceased |
| 89 | Metastatic | N | Deceased |
| 102 | Metastatic | N | Deceased |

**Table S6.** PDAC patients’ disease progression and treatment.

| **Pt ID** | **Age** | **Cancer type** | **Time to progression (months)** | **CA9-19 (U/ml)** | **Preoperative chemotherapy/ radiation** | **Postoperative chemotherapy/ radiation** | **Metastatic sites** | **Status at last follow up** | |
| --- | --- | --- | --- | --- | --- | --- | --- | --- | --- |
| 45 | 63 | Local, resected | 9.2 | 1764 | no/no | yes/yes | liver, lung | | deceased |
| 46 | 66 | local, resected | -- | -- | no/no | yes/yes | -- | | alive, disease free |
| 48 | 44 | local, resected | 4.6 | 25 | no/no | yes/no | liver | | deceased |
| 66 | 60 | local, resected | 19.5 | 1 | yes/no | --- | liver | | deceased |
| 67 | 50 | locally advanced unresectable | 4.9 | 141 | yes/no | --- | liver, peritoneal | | deceased |
| 25 | 59 | metastatic | 0.0 | 7 | --- | --- | liver | | deceased |
| 41 | 65 | metastatic | 16.7 | 22 | yes/yes | no | liver | | deceased |

**Table S7.** Epithelial ovarian cancer (EOC) patients enrolled in the study. na not applicable

| **Pt**  **ID** | **Age** | **Date diagnosed** | **Date of the last chemo regimen** | **Analysis date** | **Initial treatment/**  **chemo regimen** | **CA125 at diagnosis** | **Preop. CA125** | **Stage/ Grade** | **Histology** | **Metastasis site** |
| --- | --- | --- | --- | --- | --- | --- | --- | --- | --- | --- |
| **L-EOC-no chemo** | | |  |  |  |  |  |  |  |  |
| #2 | 41 | 01/22/2014 | na | 01/22/14 | debulking | 123 | 123 | IA/3 | Endometrioid adenocarcinoma of the ovary | None |
| #6 | 61 | 05/12/2014 | na | 05/12/14 | debulking | 835 | 835 | IA/2 | Endometrioid adenocarcinoma of the ovary | None |
| #17 | 60 | 03/2315 | na | 04/24/15 | debulking | 12 | 12.3 | IC2/3 | Papillary serous carcinoma of the ovary | None |
| **M-EOC-chemo** | | |  |  |  |  |  |  |  |  |
| #4 | 71 | 12/19/2013 | 02/01/14 | 03/11/14 | neoadjuvant chemotherapy: Carboplatin + paclitaxel, 3 cycles/ interval debulking | 5900 | 437 | IIIC/3 | Papillary serous carcinoma  of the ovary | Omentum, fallopian tube |
| #10 | 68 | 6/25/2014 | 08/25/14 | 09/26/14 | Neoadjuvant chemotherapy: Carboplatin + paclitaxel, 3 cycles/ interval debulking | 3580 | 495 | IIIC/3 | Serous carcinoma  of the peritoneum | Omentum, ovaries, uterus, sigmoid colon, appendix |
| #12 | 53 | 04/22/2014 | 10/01/14 | 10.22/14 | Neoadjuvant chemotherapy: Carboplatin + paclitaxel, 6 cycles/ interval debulking | 1423 | 290 | IV/3 | Mixed serous, clear cell, and endometrioid adenocarcinoma of the ovary | Omentum, fallopian tube |
| #14 | 45 | 12/09/14 | 02/25/15 | 03/27/15 | Neoadjuvant chemotherapy: Carboplatin + paclitaxel, 4 cycles/interval debulking | 1340 | 1030 | IVB/3 | Serous carcinoma of the ovary | Fallopian tubes, uterus, cervix, appendix |
| #19 | 58 | 12/04/14 | 03/30/15 | 05/22/15 | Neoadjuvant chemotherapy: Carboplatin + paclitaxel, 6 cycles/interval debulking | 2390 | 18.3 | IVB/3 | Papillary serous carcinoma  of the ovary | Uterus, fallopian tubes, cervix, omentum, anterior abdominal wall |
| **M-EOC-no chemo** | | |  |  |  |  |  |  |  |  |
| #1 | 65 | 01/14/2014 | na | 01/17/14 | debulking | na | na | IIIC/  Low | Serous carcinoma  of the ovary | Omentum, peritoneum, fallopian tube, lymph nodes |
| #5 | 68 | 03/12/2014 | na | 03/12/14 | debulking | 281 | 281 | IIA/3 | Carcinosarcoma  of the ovary | Fallopian tube |
| #8 | 63 | 07/25/2014 | na | 07/25/14 | debulking | 1230 | 1230 | IIIC/3 | Serous carcinoma  of the peritoneum | Omentum, pelvis, uterus, fallopian tube, ovary, sigmoid colon |
| #9 | 49 | 10/01/2009 | 09/01/11 | 09/03/14 | debulking/ Carboplatin + taxotere + Avastin, 6 cycles, then PARP inhibitor trial x 15 courses (recurrence 7/8/14)/ secondary debulking | 355 | 4.5 | IIIC/3 | Papillary serous carcinoma  of the ovary | Sigmoid colon |
| #11 | 62 | 10/03/2014 | na | 10/03/14 | debulking | 154 | 154 | IIIC/3 | Papillary serous carcinoma of the fallopian tube | Omentum, ovaries |
| #13 | 66 | 11/06/2014 | na | 11/06/14 | debulking | 915 | 915 | IIIC/3 | Papillary serous carcinoma of the ovary | Omentum, fallopian tube, peritoneum, spleen |
| #18 | 66 | 04/24/15 | na | 05/01/15 | debulking | 1500 | 1500 | IIIC/3 | Papillary serous carcinoma of the ovary | Omentum, small bowel mesentery, appendix, rectum |
| #20 | 70 | 05/14/15 | na | 05/28/15 | debulking | 296 | 296 | IIIC/3 | Papillary serous carcinoma of the ovary | terus, fallopian tubes, omentum, peritoneum, colon, small bowel mesentery, appendix, abdominal wall |

**Table S8.** CRPC patients enrolled in the study.

| **Patient ID** | **Metastatic sites** | **Treatment** | **PSA (ng/ml)** |
| --- | --- | --- | --- |
| **1** | none | ADT*, bicalutamide, abiraterone | 0.6 |
| **2** | bone, liver | ADT, abiraterone, docetaxel | 363 |
| **3** | bone | ADT, ketoconazole, sipuleucel-T | 6.4 |
| **4** | bone | ADT, docetaxel, abiraterone | 0.7 |
| **5** | bone | ADT, bicalutamide, docetaxel | 1920 |

*ADT- androgen deprivation therapy

**Table S9.** Comparison of CTC numbers secured via phenotyping (stain) and impedance sensing (e-count).

| **Pt ID** | **FAPα Selection Chip** | | | | **EpCAM Selection Chip** | | | |
| --- | --- | --- | --- | --- | --- | --- | --- | --- |
|  | **WBC/ml via stain** | **CTC^FAPα^ /ml via**  **stain** | **CTC^FAPα^ /ml via**  **e-count** | **%RSD between staining and e-count** | **WBC/ml via stain** | **CTC EpCAM /ml via stain** | **CTC EpCAM /ml via**  **e-count** | **%RSD between staining and e-count** |
| #24 | 2 | 21 | 24.5 | 10.9 | 3 | 23.5 | n/a | n/a |
| #25 | 3 | 9.5 | 6.5 | 26.5 | 2 | 23.0 | 21.0 | 8.3 |
| #46 | 1 | 7.5 | 4.0 | 43.0 | 2 | 4.0 | 4.5 | 12.5 |
| #66 | 3 | 25.0 | 34.5 | 22.5 | 5 | 52.5 | 44.0 | 29.9 |
| #68 | 4 | 16.5 | 26.0 | 31.6 | 2 | 10.0 | 6.5 | 6.4 |

*%RSD=(SD/Avg)x100. n/a = Not applicable. In this case, data was not collected.

**Table S10.** CTC enumeration data from all patients. Summary of the enumeration data for CTC^FAPα^ and CTC^EpCAM^ isolated from patients on serially connected CTC selection microfluidic devices.

| Samples  (n-number of samples,  m-number of measurements ) | CTC ^FAPα^ /ml | | | CTC ^EpCAM^ /ml | | | |  |
| --- | --- | --- | --- | --- | --- | --- | --- | --- |
|  | Average | Median | Range | Average | Median | | Range | |
| Healthy Donor  (n=11, m=11) | 0.3 | 0.0 | 0-1.0 | 0.1 | 0.0 | 0.0-0.5 | |  |
| Non-Cancer  (n=6, m=6) | 1.8±1.8* | 1.5 | 0.0-4.5 | 2.6±1.5* | 3.0 | 0.5-4.0 | |  |
| L-PDAC  (n=5, m=17) | 29.9 | 26.0 | 3.0-79.0 | 23.1 | 22.0 | 2.7-52.5 | |  |
| L-PDAC at OR  (n=3, m=6) | 31.7 | 19.7 | 16.0-59.5 | 19.1 | 13.0 | 8.7-35.5 | |  |
| M-PDAC  (n=10, m=25) | 22.1 | 17.5 | 6.5-83.3 | 27.3 | 20.5 | 3.5-105.4 | |  |
| L-CRC  (n=3, m=5) | 66.6 | 15.0 | 10.0-280.0 | 16.1 | 13.0 | 7.0-34.0 | |  |
| M-CRC  (n=3, m=4) | 34.4 | 32.8 | 26.0-48.5 | 47.8 | 31.5 | 17.0-111.0 | |  |
| M-BC  (n=10, m=12) | 41.8 | 24.0 | 0.5-179.0 | 88.7 | 47.8 | 1.0-278.0 | |  |
| M-EOC-no-chemo  (n=8, m=12) | 57.8 | 35.5 | 14.0-160.0 | 213.7 | 128.5 | 64.5-680.0 | |  |
| M-EOC-chemo  (n=5, m=10) | 27.6 | 32.0 | 4.5-42.0 | 47.9 | 42.0 | 29.0-68.0 | |  |
| L- EOC  -no chemo  (n=3, m=3) | 31.3 | 18.0 | 16.5-59.3 | 121.3 | 120.0 | 73.3-170.5 | |  |
| CRPC  (n=5, m=5) | 19.5 | 18.0 | 12.7-27.3 | 12.7 | 9.3 | 2.0 – 39.3 | |  |

*-SD used for determination of clinical sensitivity and selectivity, n-number of samples, m-number of measurements

**Table S11.** Pairwise statistical analyses of CTC counts.

| **Clinical Samples** | **p-value*** | |
| --- | --- | --- |
|  | **CTC^FAP^** | **CTC ^EpCAM^** |
| L-PDAC vs. HD (n=5, m=17) | 18.8 x10^-4^ | 0.1 x10^-4^ |
| L-PDAC at OR vs. HD (n=3, m=6) | 80.4 x10^-4^ | 22.2 x10^-4^ |
| M-PDAC vs. HD (n=10, m=25) | 0.47x10^-4^ | 0.12x10^-4^ |
| L-CRC vs. HD (n=3, m=5) | 120.8 x10^-4^ | 22.2 x10^-4^ |
| M-CRC vs. HD (n=3, m=4) | not determined** | not determined** |
| M-BC vs. HD (n=10, m=12) | 26.6 x10^-4^ | 6.7x10^-4^ |
| M-EOC-no-chemo vs. HD (n=8, m=12) | 43.8 x10^-4^ | 3.4 x10^-4^ |
| M-EOC-chemo vs. HD (n=5, m=10) | 120.8 x10^-4^ | 22.2 x10^-4^ |
| L- EOC -no chemo vs. HD (n=3, m=3) | not determined** | not determined** |
| CRPC (n=5, m=5) | 79.4x10^-4^ | 79.4x10^-4^ |
| L-PDAC vs. NC (n=5, m=17) | 13.2 x10^-4^ | 7.8 x10^-4^ |
| L-PDAC at OR vs. NC (n=3, m=6) | 38.0 x10^-4^ | 48.0 x10^-4^ |
| M-PDAC vs. NC (n=10, m=25) | 0.030x10^-4^ | 0.008x10^-4^ |
| L-CRC vs. NC (n=3, m=5) | 57.8 x10^-4^ | 43.8 x10^-4^ |
| M-CRC vs. NC (n=3, m=4) | not determined** | not determined** |
| M-BC vs. NC (n=10, m=12) | 67.8x10^-4^ | 117.0 x10^-4^ |
| M-EOC-no-chemo vs. NC (n=8, m=12) | 14.8 x10^-4^ | 9.4 x10^-4^ |
| M-EOC-chemo vs. NC (n=5, m=10) | 73.6 x10^-4^ | 43.8 x10^-4^ |
| L-EOC -no chemo vs. NC (n=3, m=3) | not determined** | not determined** |
| CRPC (n=5, m=5) | 25.2x10^-4^ | 31.0x10^-4^ |

*- p<500 x10^-4^  (<0.05) considered statistically significant

**-not enough data points for Mann-Whitney U-Test

HD- healthy donor

NC-non-cancer

**Table S12.** NGS for CTC subpopulations isolated from an ovarian cancer patient. Mutations were detected using the TruSight Tumor Sequencing Panel. Q for all = 100, GQX = 100. *- het- heterozygous, hom- homozygous. NGS data were processed using VariantStudio (Illumina).

| **Gene** | **Variant(Chr#)** | **HGVSc** | **Mut. Frequency (%)** | | **Read Depth** | | **Classification** | **Genotype** | **Consequences** |
| --- | --- | --- | --- | --- | --- | --- | --- | --- | --- |
|  |  |  | **CTC^FAPα^** | **CTC^EpCAM^** | **CTC^FAPα^** | **CTC^EpCAM^** |  |  |  |
| **PDGFRA** | A>G/G (4) | c.1701A>G | 99.8 | 99.9 | 14419 | 22908 | Synonymous protein change | hom | synonymous variant |
| **APC** | G>G/A (5) | c.4479G>A | 45.3 | 41.7 | 19408 | 25760 | Synonymous protein change | het | synonymous variant |
| **EGFR-AS1** | G>G/A (7) | n.1201C>T | 55.0 | 50.0 | 11545 | 21077 | Synonymous protein change | het | non coding exon variant and transcript variant |
| **EGFR-AS1** | G>G/A (7) | c.2361G>A | 55.0 | 50.0 | 11545 | 21077 | Synonymous protein change | het | synonymous variant |
| **MET** | G>G/A (7) | c.4071G>A | 46.3 | 50.3 | 17913 | 20455 | Synonymous protein change | het | synonymous variant |
| **MET** | G>G/A (7) | c.4146G>A | 55.2 | 54.0 | 9918 | 11573 | Synonymous protein change | het | synonymous variant |
| **CDH1** | G>G/A (16) | c.1774G>A | 58.6 | 49.2 | 46254 | 56978 | variant of uncertain significance (VUS) | het | missense variant |
| **TP53** | G>G/C (17) | c.215 C>G | 40.6 | 66.0 | 42310 | 64875 | SNP (>1% of population) | het | missense variant |

COSMIC Histology for all: carcinoma

**Table S13.** Primers sequences used for the ligase detection reactions. ph – phosphorylated

| **Mutation** | **Discriminating primer 5’ - 3’** | **Common primer 5’ – 3’** | **Ligation product size (nt)** |
| --- | --- | --- | --- |
| **35 WT** | TTTTTTTAAACTTGTGGTAGTTGGAGCTGG (30 nt) | ph-TGGCGTAGGCAAGAGTGCCT-Cy5 (20 nt) | **50** |
| **G35A** | TAAACTTGTGGTAGTTGGAGCTGA (24 nt) |  | **44** |
| **G35T** | TTTTTTTTTTTTTAAACTTGTGGTAGTTGGAGCTGT  (35 nt) |  | **55** |
| **34 WT** | TTTTTTTTTTTTTTTAAACTTGTGGTAGTTGGAGCTG (37 nt) | ph-GTGGCGTAGGCAAGAGTGCCTTGACGATAC-Cy5 (30nt) | **67** |
| **G34C** | TTTTTTTTTAAACTTGTGGTAGTTGGAGCTC (31 nt) |  | **61** |

**Table S14.** The average number of CTC^FAPα^ and CTC^EpCAM^ isolated from the blood of metastatic breast ductal carcinoma patients (M-BC). n.a., not applicable, as cells were enumerated with the impedance detector. Also shown is the number of white blood cells enumerated for each assay.

| Patient Id | Analysis date | Blood volume (ml) | Affinity bed | CTC/blood volume | WBC/blood volume | Purity (%) | CTC/ml |
| --- | --- | --- | --- | --- | --- | --- | --- |
| #1 | 11/14/13 | 2 | FAPα | 1 | 2 | 33.3 | 0.5 |
| #1 | 11/14/13 | 2 | FAPα | 1 | 0 | 100 | 0.5 |
| #1 | 11/14/13 | 2 | EpCAM | 3 | 0 | 100 | 1.5 |
| #1 | 11/14/13 | 2 | EpCAM | 2 | 2 | 50 | 1 |
| #1 | 11/14/13 | 2 | IgG bed | 0 | 2 | 0 | 0 |
| #3 | 11/14/13 | 2 | FAPα | 6 | 2 | 75 | 3 |
| #3 | 11/14/13 | 2 | EpCAM | 2 | 0 | 100 | 1 |
| #4 | 11/21/13 | 2 | FAPα | 115 | 3 | 97.4 | 57.5 |
| #4 | 11/21/13 | 2 | EpCAM | 468 | 2 | 99.6 | 234 |
| #4 | 11/21/13 | 2 | pristine COC | 0 | 0 | na | 0 |
| #5 | 12/05/13 | 2 | FAPα | 358 | 5 | 98.6 | 179 |
| #5 | 12/05/13 | 2 | FAPα | 418 | 6 | 98.6 | 209 |
| #5 | 12/05/13 | 2 | EpCAM | 556 | 3 | 99.5 | 278 |
| #5 | 12/05/13 | 2 | EpCAM | 472 | 2 | 99.6 | 236 |
| #2 | 12/12/13 | 2 | FAPα | 57 | 5 | 91.9 | 28.5 |
| #2 | 12/12/13 | 2 | EpCAM | 81 | 4 | 95.3 | 40.5 |
| #6 | 01/16/14 | 2 | FAPα | 12 | na | na | 6 |
| #6 | 01/16/14 | 2 | EpCAM | 9 | na | na | 4.5 |
| #7 | 01/16/14 | 2 | FAPα | 21 | na | na | 10.5 |
| #7 | 01/16/14 | 2 | EpCAM | 7 | na | na | 3.5 |
| #8 | 01/23/14 | 2 | FAPα | 124 | na | na | 62 |
| #8 | 01/23/14 | 2 | EpCAM | 110 | na | na | 55 |
| #9 | 01/23/14 | 2 | FAPα | 39 | na | na | 19.5 |
| #9 | 01/23/14 | 2 | EpCAM | 190 | na | na | 95 |
| #9 | 01/23/14 | 2 | IgG | 3 | 9 | na | na |
| #10 | 01/23/14 | 2 | FAPα | 103 | na | na | 51.5 |
| #10 | 01/23/14 | 2 | EpCAM | 347 | na | na | 173.5 |

**Table S15.** The average number of CTC^FAPα^ and CTC^EpCAM^ isolated per ml of blood for metastatic pancreatic ductal adenocarcinoma (PDAC) patients. n.a., not applicable, as cells were enumerated with the impedance detector. Also shown is the number of white blood cells (WBCs) enumerated per each assay as well as the calculated purity.

| **Patient ID** | **Analysis date** | **Affinity bed** | **Blood volume (ml)** | **CTC/ml** | **WBC/ml** | **Purity (%)** |
| --- | --- | --- | --- | --- | --- | --- |
| 24 | 08/07/12 | FAPα | 2 | 21.0 | 2 | 91.3 |
|  |  | EpCAM | 2 | 23.5 | 3 | 88.7 |
| 2 | 08/17/12 | FAPα | 2 | 8.5 | 0.5 | 94.4 |
|  |  | FAPα | 2 | 14.5 | 3 | 82.8 |
|  |  | EpCAM | 2 | 6.5 | 2 | 76.5 |
|  |  | EpCAM | 2 | 3.5 | 3 | 53.8 |
| 42 | 08/27/12 | FAPα | 1.5 | 83.3 | 4 | 95.4 |
|  |  | EpCAM | 2 | 49 | 2 | 96.1 |
| 40 | 08/31/12 | FAPα | 2 | 21 | 2.5 | 89.4 |
|  |  | FAPα | 2 | 12.5 | 1 | 92.6 |
|  |  | EpCAM | 2 | 31.5 | 1 | 96.9 |
|  |  | EpCAM | 2 | 77.5 | 4 | 95.1 |
| **25** | 09/21/12 | FAPα | 3 | 29.3 | na | na |
|  |  | FAPα | 3 | 30.7 | na | na |
|  |  | EpCAM | 3 | 19 | na | na |
|  |  | EpCAM | 3 | 18 | na | na |
| 47 | 09/28/12 | FAPα | 2 | 24.5 | na | na |
|  |  | FAPα | 2 | 38.5 | na | na |
|  |  | EpCAM | 2 | 20 | na | na |
|  |  | EpCAM | 2 | 9.5 | na | na |
| 41 | 11/16/12 | FAPα | 2 | 25.5 | na | na |
|  |  | FAPα | 2 | 15.5 | na | na |
|  |  | EpCAM | 2 | 105 | na | na |
|  |  | EpCAM | 2 | 60 | na | na |
| **25** | 11/29/12 | FAPα | 2 | 9.5 | 2 | 82.6 |
|  |  | FAPα | 2 | 6.5 | na | na |
|  |  | EpCAM | 2 | 23 | 3 | 88.5 |
|  |  | EpCAM | 2 | 21 | na | na |
| **25** | 02/07/13 | FAPα | 2 | 10 | 3 | 76.9 |
|  |  | FAPα | 2 | 7.5 | 2.5 | 75.0 |
|  |  | EpCAM | 2 | 20 | 3 | 86.9 |
|  |  | EpCAM | 2 | 34.5 | 2 | 94.5 |
| **25** | 03/21/13 | FAPα | 2 | 14 | na | na |
|  |  | EpCAM | 2 | 5.5 | na | na |
| 41 | 03/22/13 | FAPα | 2 | 13.5 | na | na |
|  |  | EpCAM | 2 | 16 | na | na |
|  |  | EpCAM | 2 | 39 | na | na |
| 68 | 04/19/13 | FAPα | 2 | 26 | na | na |
|  |  | FAPα | 2 | 16.5 | 4 | 80.5 |
|  |  | EpCAM | 2 | 4.5 | na | na |
|  |  | EpCAM | 2 | 6.5 | na | na |
|  |  | EpCAM | 2 | 10 | 2 | 83.3 |
| 102 | 05/02/13 | FAPα | 2 | 40 | na | na |
|  |  | EpCAM | 2 | 23.5 | na | na |
| 48 | 12/14/12 | FAPα | 2 | 19 | na | na |
|  |  | FAPα | 2 | 34.5 | na | na |
|  |  | EpCAM | 2 | 25 | na | na |
|  |  | EpCAM | 2 | 19.5 | na | na |

**Table S16.** The average number of CTC^FAPα^ and CTC^EpCAM^ isolated per ml of blood for localized pancreatic ductal adenocarcinoma (PDAC) patients.

| **Patient ID** | **Analysis date** | **Affinity bed** | **Blood volume (ml)** | **CTC/ml** |
| --- | --- | --- | --- | --- |
| **45 OR** | 09/20/12 | FAPα | 3 | 16.3 |
|  |  | FAPα | 3 | 23.3 |
|  |  | EpCAM | 3 | 15.3 |
|  |  | EpCAM | 3 | 8.3 |
| 45 | 12/13/12 | FAPα | 2 | 25.5 |
|  |  | EpCAM | 2 | 18.5 |
| 45 | 02/28/13 | FAPα | 2 | 7.5 |
|  |  | FAPα | 2 | 4.0 |
|  |  | EpCAM | 2 | 9.0 |
|  |  | EpCAM | 2 | 9.5 |
| 45 | 04/15/13 | FAPα | 2 | 17.5 |
|  |  | FAPα | 2 | 19.5 |
|  |  | EpCAM | 2 | 5 |
|  |  | EpCAM | 2 | 8.5 |
| **46 at OR** | 09/20/12 | FAPα | 3 | 13.7 |
|  |  | FAPα | 3 | 25. 7 |
|  |  | EpCAM | 3 | 8. 7 |
|  |  | EpCAM | 3 | na |
| 46 | 10/08/12 | FAPα | 2 | 3.5 |
|  |  | FAPα | 2 | 7 |
|  |  | EpCAM | 2 | 3 |
|  |  | EpCAM | 2 | 5 |
| 46 | 11/12/12 | FAPα | 1.5 | 3.3 |
|  |  | FAPα | 1.5 | 2. 7 |
|  |  | EpCAM | 1.5 | 4.0 |
|  |  | EpCAM | 1.5 | 1.3 |
| 46 | 06/03/13 | FAPα | 2 | 17 |
|  |  | FAPα | 2 | 12 |
|  |  | EpCAM | 2 | 20.5 |
|  |  | EpCAM | 2 | 32 |
| **66 at OR** | 12/06/12 | FAPα | 2 | 23 |
|  |  | FAPα | 2 | 9 |
|  |  | EpCAM | 2 | 15.5 |
|  |  | EpCAM | 2 | 10.5 |
| 66 | 03/11/13 | FAPα | 2 | 34.5 |
|  |  | FAPα | 1 | 34 |
|  |  | FAPα | 2 | 25 |
|  |  | EpCAM | 2 | 44 |
|  |  | EpCAM | 2 | 52.5 |
|  |  | EpCAM | 1 | 57 |
| 66 | 06/17/13 | FAPα | 3 | 99.3 |
|  |  | FAPα | 3 | 58.7 |
|  |  | EpCAM | 3 | 55.0 |
|  |  | EpCAM | 3 | 50.0 |
| **48** | 05/23/12 | FAPα | 2 | 26 |
|  |  | EpCAM | 2 | 11.5 |
| 48 | 12/14/12 | FAPα | 2 | 19 |
|  |  | FAPα | 2 | 34.5 |
|  |  | EpCAM | 2 | 25 |
|  |  | EpCAM | 2 | 19.5 |
| **67** | 03/22/13 | FAPα | 2 | 20 |
|  |  | EpCAM | 2 | 28 |

**Table S17.** The average number of CTC^FAPα^ and CTC^EpCAM^ isolated from the blood of colorectal cancer patients. nd – not determined (impedance measurements). Also shown is the blood volume analyzed and the average number of white blood cells (WBCs) enumerated.

| Patient ID | Cancer Type | Analysis date | Affinity bed | Blood volume processed (ml) | CTC/ml | WBCs/ml | Purity (%) |
| --- | --- | --- | --- | --- | --- | --- | --- |
| 100 | Colorectal, metastatic | 04/24/12 | FAPα | 2 | 48.5 | nd | nd |
|  |  |  | FAPα | 2 | 26 | nd | nd |
|  |  |  | EpCAM | 2 | 18 | nd | nd |
|  |  |  | EpCAM | 2 | 45 | nd | nd |
| 118 | Colorectal, metastatic | 05/02/13 | FAPα | 2 | 39.5 | nd | nd |
|  |  |  | EpCAM | 2 | 111 | nd | nd |
| 125 | Rectal, metastatic | 05/03/13 | FAPα | 2 | 23.5 | nd | nd |
|  |  |  | EpCAM | 2 | 17 | nd | nd |
| 116 | Colorectal, non-metastatic | 05/01/13 | FAPα | 2 | 16 | nd | nd |
|  |  |  | FAPα | 2 | 12 | nd | nd |
|  |  |  | EpCAM | 2 | 14 | nd | nd |
|  |  |  | EpCAM | 2 | 11.5 | nd | nd |
| 122 | Rectal,  non-metastatic | 05/03/13 | FAPα | 2 | 15 | nd | nd |
|  |  |  | FAPα | 2 | 10 | nd | nd |
|  |  |  | EpCAM | 2 | 13 | nd | nd |
|  |  |  | EpCAM | 2 | 7 | nd | nd |
| 135 | Colorectal, non-metastatic | 06/14/13 | FAPα | 2.5 | 280 | 7.2 | 97.4 |
|  |  |  | EpCAM | 2.5 | 34.8 | 4.8 | 87.9 |

**Table S18.** Average number of CTC^FAPα^ and CTC^EpCAM^ isolated from the blood of CRPC patients along with the purity of the selected fraction for CTC^EpCAM^.

| Patient ID | Analysis date | Blood volume processed (ml) | CTC^FAPα^/ml | CTC^EpCAM^/ml | WBC/ml in EpCAM bed | Purity (%) |
| --- | --- | --- | --- | --- | --- | --- |
| 1 | 11/15/2012 | 1.5 | 12.7 | 39.3 | 4 | 90.8 |
| 2 | 11/08/2012 | 1.5 | 18.0 | 9.3 | 8 | 53.8 |
| 3 | 11/08/2012 | 1.5 | 27.3 | 33.3 | 8.7 | 79.3 |
| 4 | 11/15/2012 | 1.5 | 16.0 | 4.0 | 4.7 | 45.9 |
| 5 | 11/19/2012 | 1.5 | 23.3 | 20.0 | 2.7 | 88.1 |

**Table S19.** CTC^FAPα^ and CTC^EpCAM^ isolated from the blood of epithelial ovarian cancer (EOC) patients. The CTC number shown per ml represents the average. Also shown is the purity of the isolated fraction of CTCs and the average number of white blood cells (WBCs) enumerated per assay.

| **Patient Id** | **Disease/Treatment** | **Analysis date** | **Affinity bed** | **Blood volume (ml)** | **CTC/ml** | **WBC/ml** | **Purity (%)** |
| --- | --- | --- | --- | --- | --- | --- | --- |
| #1 | M-EOC-no chemo | 01/17/14 | FAPα | 2 | 48.5 | 6 | 88.9 |
|  |  |  | EpCAM | 2 | 157 | 16 | 90.8 |
| #2 | L-EOC-no chemo | 01/22/14 | FAPα | 1.5 | 59.3 | 1.3 | 97.8 |
|  |  |  | EpCAM | 1.5 | 73.3 | 2.7 | 96.5 |
| #4 | M-EOC-chemo | 03/11/14 | FAPα | 2 | 4 | na |  |
|  |  |  | FAPα | 2 | 5 | 1 | 83.3 |
|  |  |  | EpCAM | 2 | 45 | na |  |
|  |  |  | EpCAM | 2 | 40 | 2 | 95.2 |
| #5 | M-EOC-no chemo | 03/12/14 | FAPα | 2 | 31 | 3.5 | 89.9 |
|  |  |  | EpCAM | 2 | 246 | 4.5 | 97.6 |
| #6 | L-EOC-no chemo | 05/12/14 | FAPα | 2 | 16.5 | 2.5 | 86.8 |
|  |  |  | EpCAM | 2 | 170.5 | 8 | 95.5 |
| #8 | M-EOC-no chemo | 07/25/14 | FAPα | 1 | 20 | na | na |
|  |  |  | EpCAM | 1 | 100 | na | na |
| #9 | M-EOC-no chemo | 09/03/14 | FAPα | 1.5 | 118 | na | na |
|  |  |  | EpCAM | 1.5 | 76.7 | na | na |
| #10 | M-EOC-chemo | 09/26/14 | FAPα | 1 | 42 | na | na |
|  |  |  | FAPα | 1 | 68 | na | na |
|  |  |  | EpCAM | 1 | 397 | na | na |
|  |  |  | EpCAM | 1 | 476 | na | na |
| #11 | M-EOC-no chemo | 10/03/14 | FAPα | 1 | 137 | na | na |
|  |  |  | EpCAM | 1 | 680 | na | na |
|  |  |  | FAPα | 1 | 94 | na | na |
|  |  |  | EpCAM | 1 | 943 | na | na |
| #12 | M-EOC-chemo | 10/22/14 | FAPα | 1 | 17 | na | na |
|  |  |  | EpCAM | 1 | 54 | na | na |
|  |  |  | FAPα | 1 | 26 | na | na |
|  |  |  | EpCAM | 1 | 27 | na | na |
| #13 | M-EOC-no chemo | 11/06/14 | FAPα | 2 | 32.5 | na | na |
|  |  |  | EpCAM | 2 | 69 | na | na |
|  |  |  |  |  |  |  |  |
| #14 | M-EOC-chemo | 03/27/15 | FAPα | 2 | 32 | 2 | 94.1 |
|  |  |  | EpCAM | 2 | 29 | 3 | 90.6 |
|  |  |  |  |  |  |  |  |
| #17 | L-EOC-no chemo | 04/27/15 | FAPα | 2 | 17.5 | 3 | 85.4  96.0 |
|  |  |  | EpCAM | 2 | 119.5 | 5 | 96.0 |
|  |  |  |  |  |  |  |  |
| #18 | M-EOC-no chemo | 05/01/15 | FAPα | 2 | 38 | 5 | 88.4 |
|  |  |  | EpCAM | 2 | 64.5 | 3 | 95.6 |
|  | M-EOC-chemo |  |  |  |  |  |  |
| #19 |  | 05/22/15 | FAPα | 2 | 37.8 | 3 | 92.6 |
|  |  |  | EpCAM | 2 | 59.6 | 2 | 96.7 |
|  |  |  |  |  |  |  |  |
| #20 | M-EOC-no chemo | 05/28/15 | FAPα | 2 | 13.5 | 3 | 81.8 |
|  |  |  | EpCAM | 2 | 215.5 | 4 | 98.2 |

**References**

1 Adams, A. A. *et al.* Highly efficient circulating tumor cell isolation from whole blood and label-free enumeration using polymer-based microfluidics with an integrated conductivity sensor. *Journal of the American Chemical Society* **130**, 8633-8641, doi:10.1021/ja8015022 (2008).

2 Kamande, J. W. *et al.* Modular Microsystem for the Isolation, Enumeration, and Phenotyping of Circulating Tumor Cells in Patients with Pancreatic Cancer. *Analytical Chemistry* **85**, 9092-9100, doi:10.1021/ac401720k (2013).
